# An experimental study on double longitudinal plate connection for steel T-joints subject to out-of-plane moments

**DOI:** 10.1038/s41598-025-18019-5

**Published:** 2025-09-12

**Authors:** Ahmed A. Ramzi, Ahmed A. Matloub, Ahmed H. Yousef

**Affiliations:** Department of Structural Engineering, Ain-Shams University, Cairo, Egypt

**Keywords:** Square hollow section, Experimental investigation, Out-of-plane moment, Double longitudinal plates, Face plastification, Sidewall buckling, Civil engineering, Metals and alloys

## Abstract

Square hollow section (SHS) is widely used as a chord member in steel trusses. In one connection type, a brace using I-shape or box section at a T-joint transfers out-of-plane bending moment to these chords through double longitudinal plates. Design codes include rules for different connections to hollow sections, but not specifically for this detail. An experimental program is presented in this paper to verify relevant design rules and apply them to the double-plate connection, considering three key parameters: double-plate spacing, connection offset from the chord centerline, and plate width. The test results are evaluated by discussing the connection capacity and the observed failure modes, in addition to plotting both the load-displacement and moment-rotation curves. Applying relative rules from code is found to be on the conservative side and requires adjustment to apply to the current context. However, the rules for single plates are found to be adequate for some of the eccentric double-plate connections with a plate at the chord face center. Some modifications to the existing design rules are proposed to suit the eccentric connection with a plate not at the center.

## Introduction

Hollow steel sections (HSS) are commonly used in modern steel structures due to their aesthetic appeal and ability to sustain different straining actions. These tubular sections are typically used in trusses as chord members, where they receive connections from I- or tube sections called hereafter as a brace, which serve as floor beams on the horizontal plane or vertical members on the truss plane. In some cases, these braces of I- or tube sections should be rigidly connected to the truss chord for stability, thus transferring their out-of-plane bending moment to the chord. Connections of the I- or tube brace section to the chord HSS are typically made by welding the flanges and webs directly to the HSS. Alternatively, the connection can be made by welding double longitudinal plates to the chord, and then bolting the flanges of the I- or tube section to these double plates. The last connection is the subject of this paper, specifically with a square hollow section (SHS) for the chord.

Other connections to the truss chord of HSS can be made from a single longitudinal plate welded to one face, or through-plate penetrating the chord to the other face. The failure mode in this type of connection is mainly chord face plastification. The stress concentration at the mid-depth of the chord face due to the tension or compression action from the plate reduces the capacity of such a connection. Rules for estimating connection capacity using yield-line theory and experimental investigations are available in the literature^1^. To enhance the connection capacity, double longitudinal plates subject to normal force are investigated instead of a single plate. The double plates exhibit higher capacity^2–5^. For T- and X-joints for HSS-to-HSS connections, the failure modes show a mix of chord face plastification and sidewall buckling or yielding. In some cases, local failure in brace or shear punching occurs depending on the dimension of the brace to the chord. Many key parameters of the truss chord affect the connection capacity, such as end distance, wall slenderness, yield strength, and axial preloading, in addition to the brace width -to- chord width ratio^6–12^. The brace is mostly connected concentrically to the chord face. However, eccentric HSS-to-HSS connections are also investigated, deriving a new developed yield line function for the brace width -to- chord width ratio^13,14^.

While much research is conducted on in-plane and axial loading of HSS connections, studies focusing on out-of-plane loading moment in HSS moment connections remain limited. The out-of-plane moment on square HSS connections is examined experimentally on X-joints between hollow sections, encompassing traditional square bird-beam and diamond bird-beak configurations. The ultimate strengths of bird-beak HSS X-joints under out-of-plane bending are generally much higher than those of conventional HSS X-joints. Available design rules in the codes are found to be on the conservative side^15^. In another study, the moment-rotation behavior of eccentric hollow section cross-type under out-of-plane moment is investigated. Chord wall plastification is the dominant failure mode, and the connection can be classified as rigid joint resistance depending on chord wall slenderness^16,17^. A reinforced square HSS X-joints are studied using collar or doubler plates, showing that the thickness of the plates affects the ultimate resistance^18^.

Despite the advances in modeling and experimental testing of in-plane and axial loading conditions, research on the behavior of such connections under out-of-plane loading is still ongoing. Proper design recommendations for this type of connection are not yet available in design codes and guidelines^19–22^. To fill this gap, an experimental program is designed in this paper to investigate the static behavior of double plates to a square hollow section (SHS) as a T-joint subject to out-of-plane bending moment.

## Experimental program

Several methods can be used to study the behavior of a steel member or connection. Computers are now widely used in practically all branches of engineering for the analysis of structures, solids, and fluids. One of these methods is the finite element analysis, an important research pillar^4,7,11,16,23^. Another method is machine learning, which has been recently employed in different structural applications^24–28^. However, laboratory tests are still the most preferred option in any field of research, presenting reality, which is what this paper used.

**Fig. 1:**
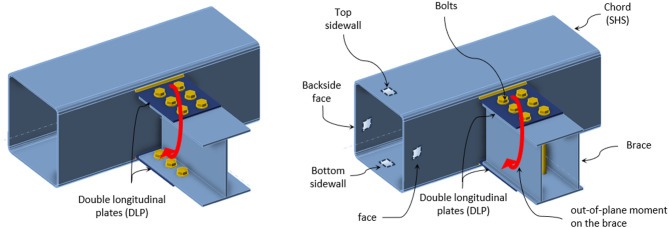
SHS connection subject to out-of-plane moment through DLP.

An experimental program is performed on specimens having double longitudinal plates. Figure 1 shows possible connections comprised of double longitudinal plates (DLP) welded to one face of the chord member. The chord member is made from a square hollow section (SHS) and receives an out-of-plane bending moment applied on the brace. This is a torsional moment on the chord, translated in the double longitudinal plates as a tension force in one plate (the top one) and a compression force in the other plate (the bottom one). The brace member can be I-profile or box section made from welded built-up plates and connected to the chord through the DLP. In the following sections, the experimental program, including the specimens’ configuration, test setup, and material properties, is illustrated. The experimental program is performed on the DLP welded to the SHS chord member, and connecting a brace box section using through bolts, as shown in Fig. 1 (right-hand side).

### Specimen configuration

A total of thirteen (13) specimens are tested in the program. The tests include seven centric connections with symmetric locations of the welded plates around the longitudinal center line of the chord. The welded plates in the remaining six specimens are eccentric with an offset toward the top or bottom sidewall of the SHS section. The investigated parameters in this study are the spacing of the double plates, their width, and their offset from the chord center line.

The SHS chord has an equal width and depth (b_0_) of 150 mm, commonly used in the field. The chord thickness (t_0_) is selected equal to 4.5 mm, with a width-to-thickness ratio less than the slender section limitation according to AISC 360 − 22^20^, where (b_0_ – 4t_0_)/t_0_ = 29.33 is less than the limit of 1.4(E/F_y_)^0.5^, assuming high-grade steel. The fabricated length of the chord is taken as constant for all specimens and equals 1200 mm. This length results in sufficiently large length-to-depth ratios for the chord to minimise the influence of the chord’s end constraint on the connection zone. This can be achieved by taking the chord length equal to or greater than the width of the brace’s connection plus three times this width from each side toward the chord ends^29,30^. To produce an out-of-plane moment on the brace, an eccentric load is used. A brace is positioned horizontally and perpendicular to the chord, having a fabricated length of 630 mm, and is vertically loaded at the tip with a concentrated load. This length is chosen to be in the same range as previous studies^31,32^, also to respect the location of the hydraulic jack and the test setup in the lab.

To limit the failure mode to the chord member only and prevent any other possible failure mode in the brace, a stiff brace is used. The brace is fabricated as a closed built-up box section to avoid lateral-torsional buckling. The brace is made from thick plates of 10 mm for flanges and webs, more than double the chord thickness, to avoid local buckling or block shear rupture in the brace. The brace is chosen with a constant width, as long as it is not a parameter, using 150 mm, while the parameter of different widths is considered in the plate not the brace. The depth of the brace is chosen variable accommodating the different examined spacings between the DLP. The brace is attached to the DLP using six high-strength bolts having a diameter (d) of 16 mm and made from high-grade steel A325M, which is characterized by yield and ultimate strengths of 630 and 880 MPa, respectively. The number, diameter, and material grade of the bolts are designed to have sufficient strength, avoiding any failure mode in the connection before the chord. The thickness of DLP (t_1_) is taken equal to 10 mm, similar to the brace, thick enough to avoid tearing or bearing failure or block shear rupture at the bolt holes. The double plates are welded to the SHS using a single fillet weld with a leg size of 6.0 mm at the accessible locations from outside. The weld size and material are also sufficient to prevent any failure in the weld. For cases where the plate is positioned in front of the chord sidewall, a partial penetration butt weld is used between the plate and the corner fillet.

The out-to-out distance of the double plates simulates here the proposed brace depth. This distance -to- chord face depth (b₁/b_0_), represented by the ratio (β), is examined at ranges of 0.5 to 1.0. The β ratio is selected not smaller than 0.5, similar to Fan and Packer^6^, to avoid any impractical condition of very small braces to the chord. The maximum β ratio is taken as 1.0, which presents brace and chord equal depths. Additionally, two groups of DLP are considered examining the width of the welded plates by taking plate widths of 100 mm and 150 mm. This width is presented as the ratio (ɳ), defining the ratio of the welded plate width -to- the chord face depth (l₁/b_0_), taken equal to 0.667 and 1.0. The maximum ɳ is taken as 1.0, considering the case that the flange width will be equal to the depth of the brace I-section (assuming HEA or HEB European profiles); thus, the flange width will not exceed the chord depth. The minimum ɳ is taken as 0.667, having a plate width of 100 mm, which is the smallest width that can be taken respecting two lines of bolts, with bolt diameter 16 mm as used, with pitch distance of 3d and edge distance of 1.5d.

**Fig. 2:**
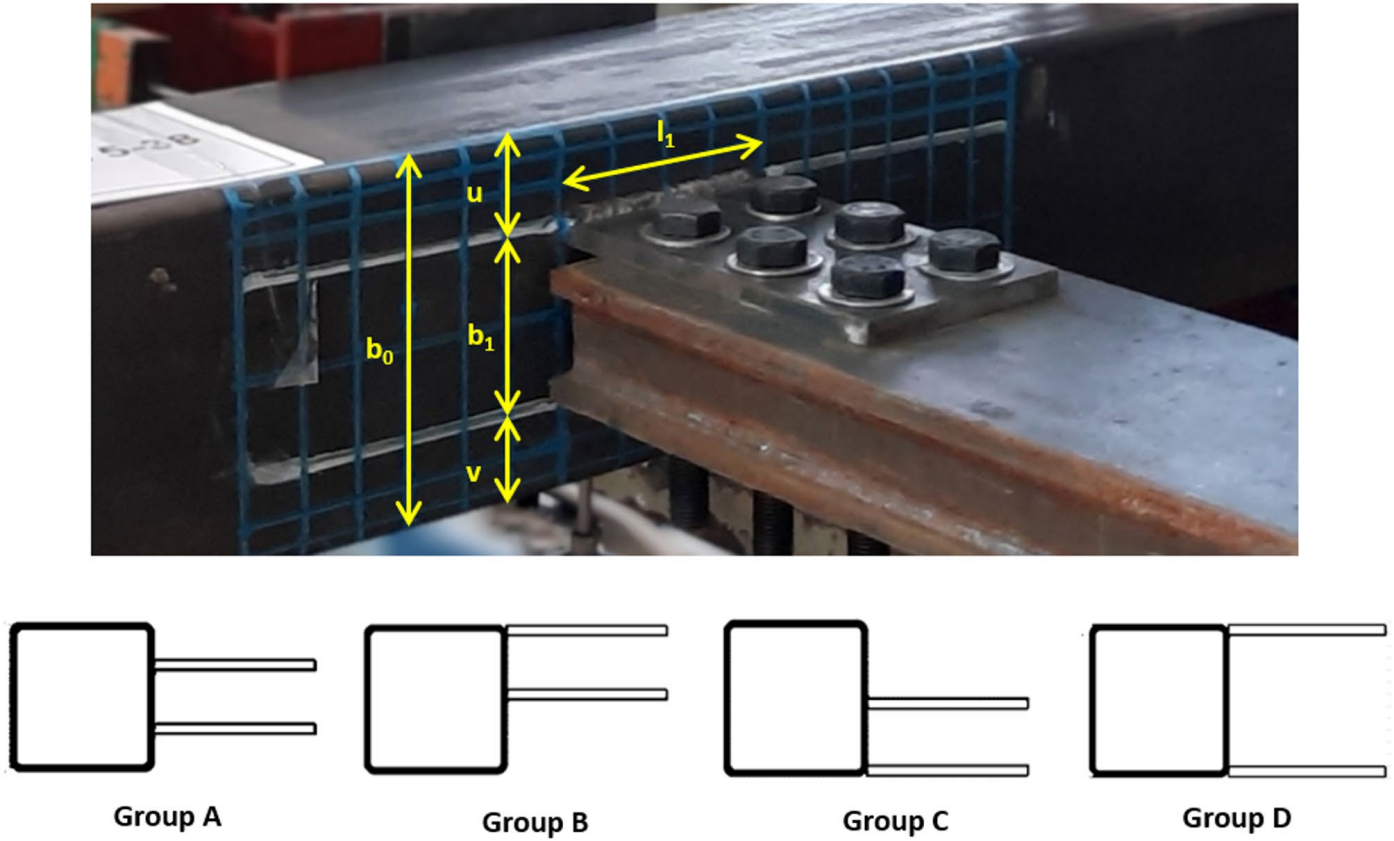
The configuration of specimens.

For easy reference, a simple labeling system is used to identify each specimen. The specimen’s label [S#-(u-b_1_-v)-l_1_] presents a series of numbers for the thirteen specimens starts from 1 to 13, followed by three values which are the distance from the top fiber of SHS’s sidewall to the top fiber of the top plate (u), then, the out-to-out distance of the double longitudinal plates (b_1_), and finally the distance from the bottom fiber of the bottom plate to the bottom fiber of SHS’s sidewall (v). At the end, the plate width l_1_ is defined. For instance, specimen S9-(18-132-0)-150 denotes that the top welded plate is located at 18 mm from the top sidewall of SHS, the outer spacing between the double plates (b_1_) is 132 mm, and the bottom welded plate is located in front of the SHS lower sidewall. The width of the DLP is 150 mm. The configuration of the specimens is illustrated in Fig. 2, and the dimensions of all specimens are listed in Table 1.

**Table 1:** The dimensions of specimens.

Identification	Connection group	β	ɳ	Eccentricity
S1-(38-75-38)-150	Group A	0.5	1.00	Centric
S2-(19-112-19)-150		0.75	1.00	Centric
S3-(9-132-9)-150		0.875	1.00	Centric
S4-(0-75-75)-150	Group B	0.5	1.00	Top offset
S5-(0-112-38)-150		0.75	1.00	Top offset
S6-(0-132-18)-150		0.875	1.00	Top offset
S7-(75-75-0)-150	Group C	0.5	1.00	Bottom offset
S8-(38-112-0)-150		0.75	1.00	Bottom offset
S9-(18-132-0)-150		0.875	1.00	Bottom offset
S10-(38-75-38)-100	Group A	0.5	0.67	Centric
S11-(19-112-19)-100		0.75	0.67	Centric
S12-(9-132-9)-100		0.875	0.67	Centric
S13-(0-150-0)-150	Group D	1.00	1.00	Centric

### Test setup

**Fig. 3:**
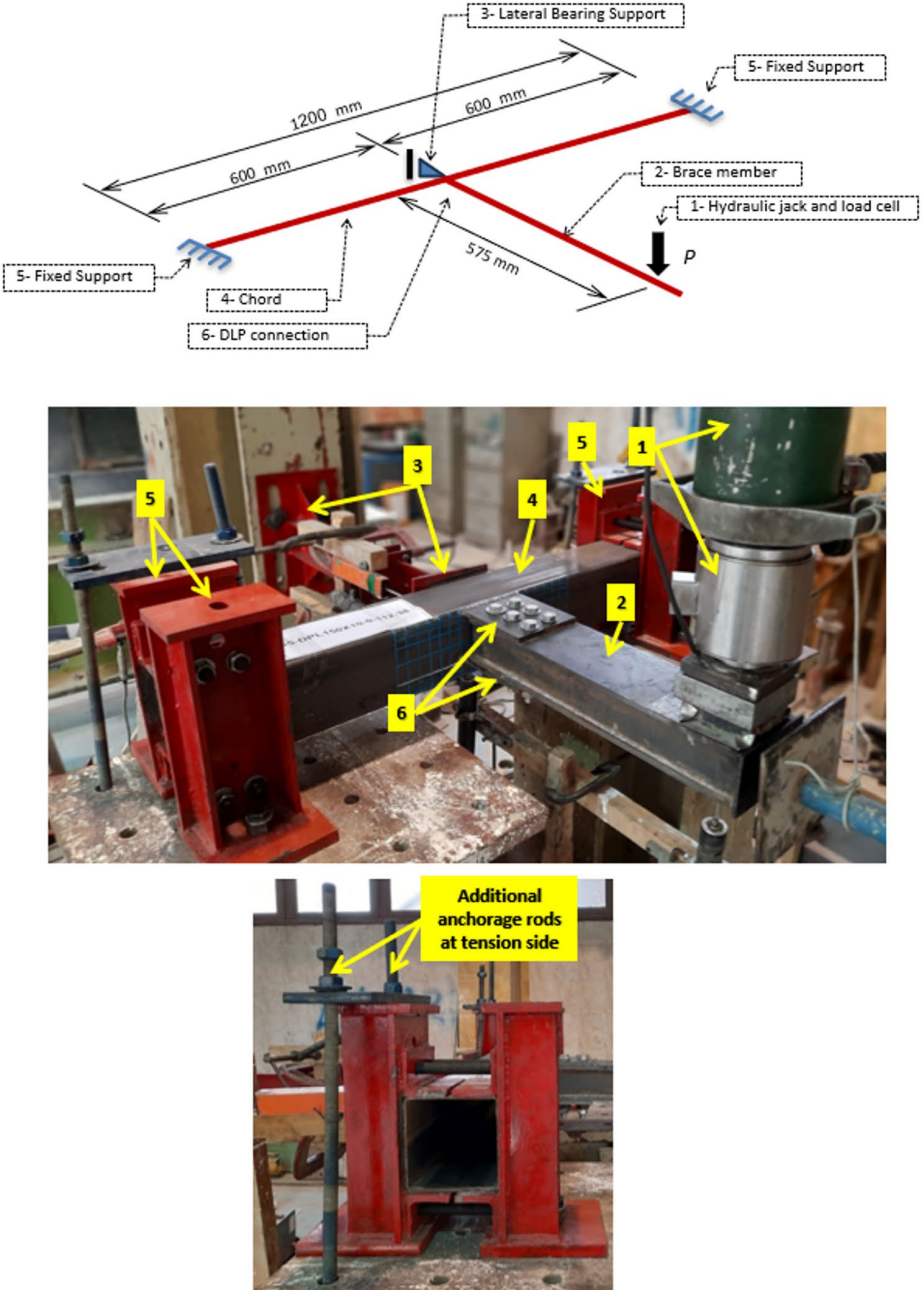
Test setup.

**Fig. 4:**
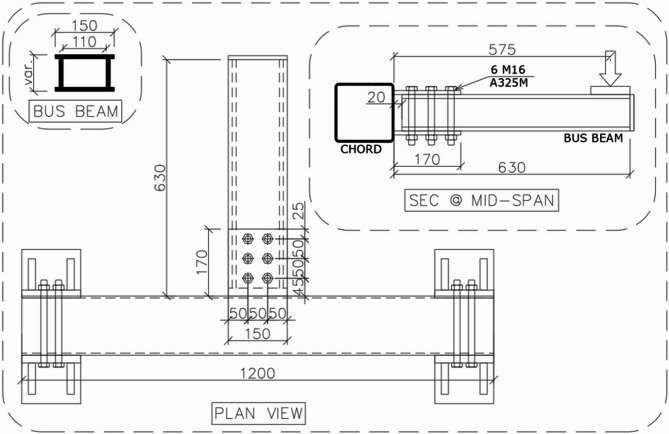
A diagrammatic sketch of the specimens.

Figure 3 shows a sketch and photo of the test setup for specimens. The SHS chord member has a length of 1200 mm with a connection exactly at the middle. The out-of-plane moment on the connection is developed by applying a static vertical load at the brace tip. The center of the loading device is at a distance of 575 mm from the SHS face. A bearing plate of thickness 30 mm is placed on the top of the brace under the load to avoid any local failure due to stress concentration, also to allow free rotation at the point of load application. The chord is rigidly supported at both ends, preventing rotation around all chord axes. The fixed support contains two separate back-to-back brackets to facilitate putting the chord inside, then connecting the brackets together through rigid bolts. The single bracket comprises of C-channel from welded plates with top and bottom bearing plates and two vertically spaced side angles back-to-back. All these items are fabricated from thick plates of 12 mm to ensure full rigidity, avoiding any local deformation. The two brackets at each support surround the chord from all sides and are connected using four high-strength through bolts, two above and two below the chord. The bolts are tightened to ensure full contact between the brackets and the chord. As the chord is mainly subject to torsional moment, additional vertical anchorages are added at the tension side of the overturning moment at the support to avoid any uplift of one bracket. Lateral bearing support is added at the chord’s mid-span, the SHS backside face, to avoid chord unbalanced drift at the connection. A diagrammatic sketch is presented in Fig. 4 for more clarification of the test setup dimensions.

**Fig. 5:**
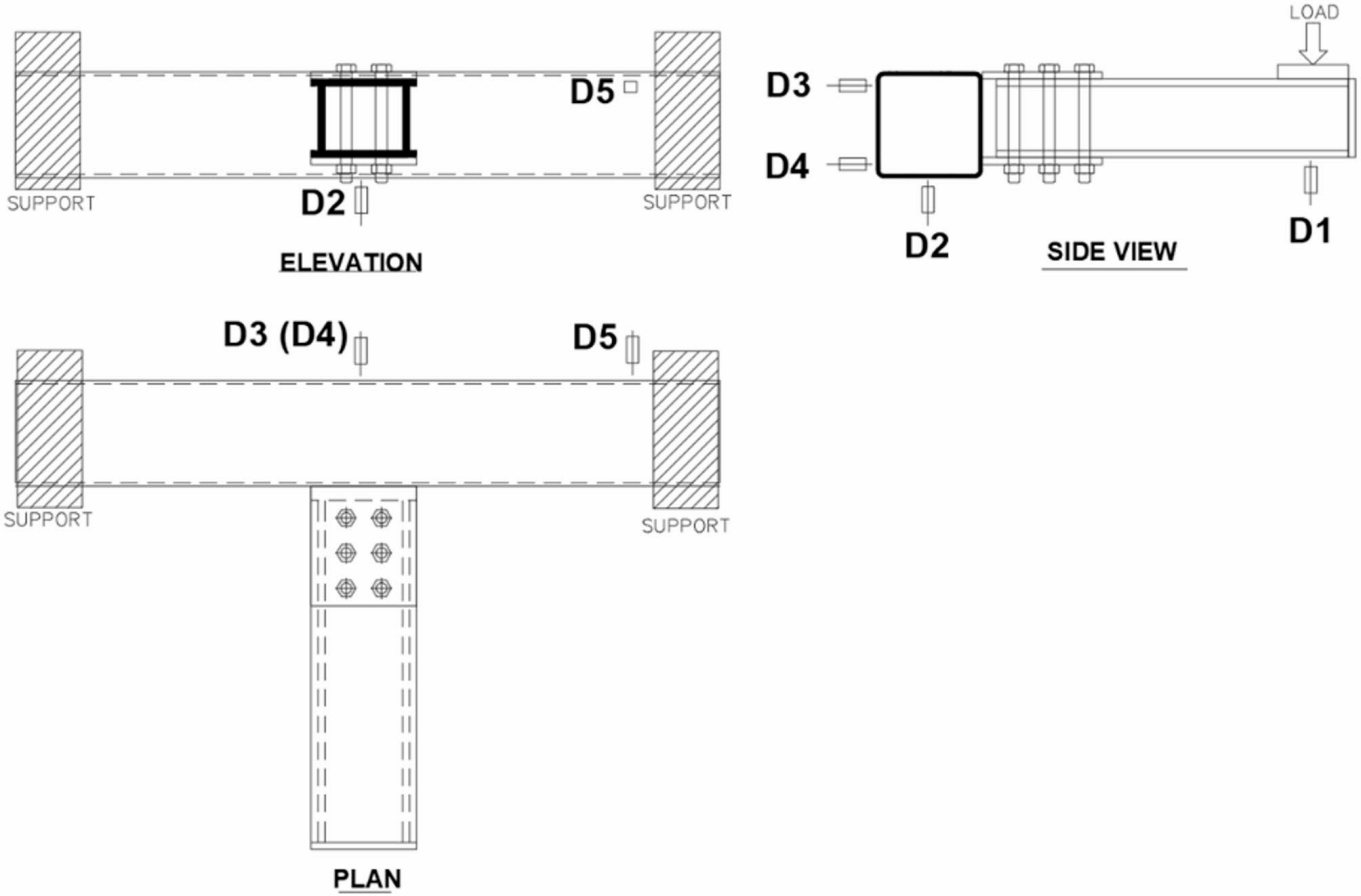
LVDTs arrangement.

Linear voltage displacement transducers (LVDTs) are utilized to measure the displacement at different locations. They are used to monitor the rotation and deformation of the chord at its midspan, also to ensure no movement of the chord supports. The arrangement of LVDT locations is shown in Fig. 5. LVDT D1 measures the vertical deflection of the brace tip under the load application, while LVDT D2 measures the vertical deflection of the chord at the connection point. D1 and D2 are used to calculate the rotation of the brace and estimate the rotation of the chord face at the connection. LVDT D3 and D4 are placed at the SHS midspan, on the chord backside face. One is located at the top point (D3), while the other is at the bottom point (D4) to measure the differential horizontal displacement of the SHS and detect any lateral deformation or torsional rotation of the chord. LVDT D5 is placed on the SHS just adjacent to the end brackets at the support point to detect any horizontal deformation and to ensure zero displacement and rotation at the support.

### Material properties

**Fig. 6:**
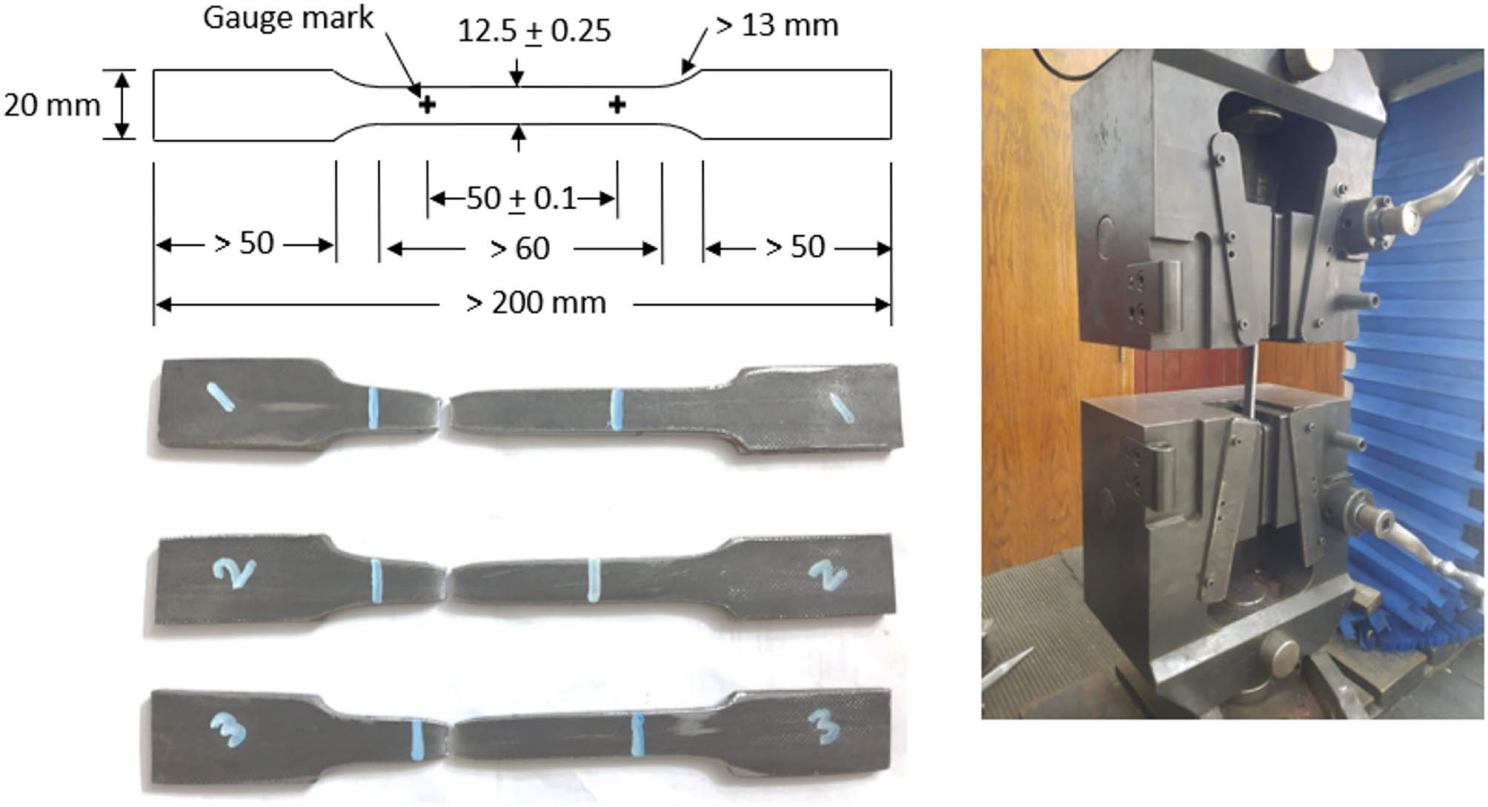
Coupons test.

**Fig. 7:**
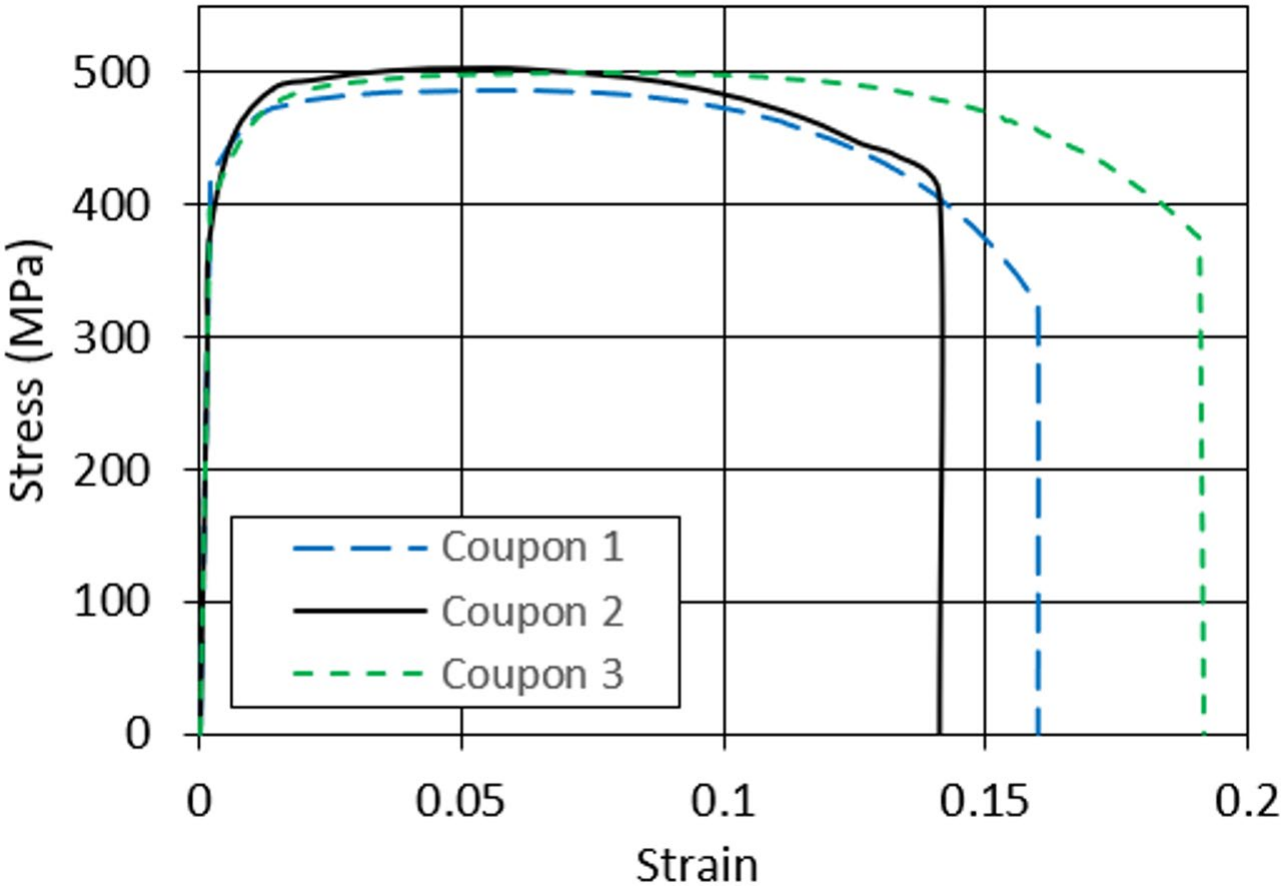
Coupons stress-strain relationship.

Tensile coupon tests are conducted to determine the stress-strain properties of the chord section. Three tensile coupons are cut from one flat plate of the SHS to assess the mechanical properties of the material. The cut part is taken in the longitudinal direction of the specimen and away from the corners. The tensile coupons are shaped and tested according to the international standards ASTM E8/E8M^33^. Using LR 300 K material strain testing machine with a 250 kN capacity, the stress and strain are recorded at regular intervals during the tests. The coupons after failure, along with the standard dimensions of the coupon and the testing machine, are shown in Fig. 6. The material mechanical properties, including yield stress (F_y_), ultimate strength (F_u_), ultimate strain (ε_u_), and modulus of elasticity (E) of the tested coupons are presented in Table 2 as average values for the three tested coupons. The stress-strain relationships are also presented in Fig. 7. The average material properties are found to be F_y_ = 400 MPa and F_u_ = 502 MPa, with elongation of 16.4%.

**Table 2:** Material properties.

	E (GPa)	F_y_ (MPa)	F_u_ (MPa)	ε_u_ (mm/mm)
Coupon 1	214	421	500	16.2
Coupon 2	216	380	505	14.8
Coupon 3	201	400	501	19.1
Average	210	400	502	16.43

## Test results

This section presents the results of the conducted experiments. The failure mode of each specimen is illustrated at the beginning. Load-deflection curves are presented after, followed by the relative moment-rotation curves.

### Failure modes

**Fig. 8:**
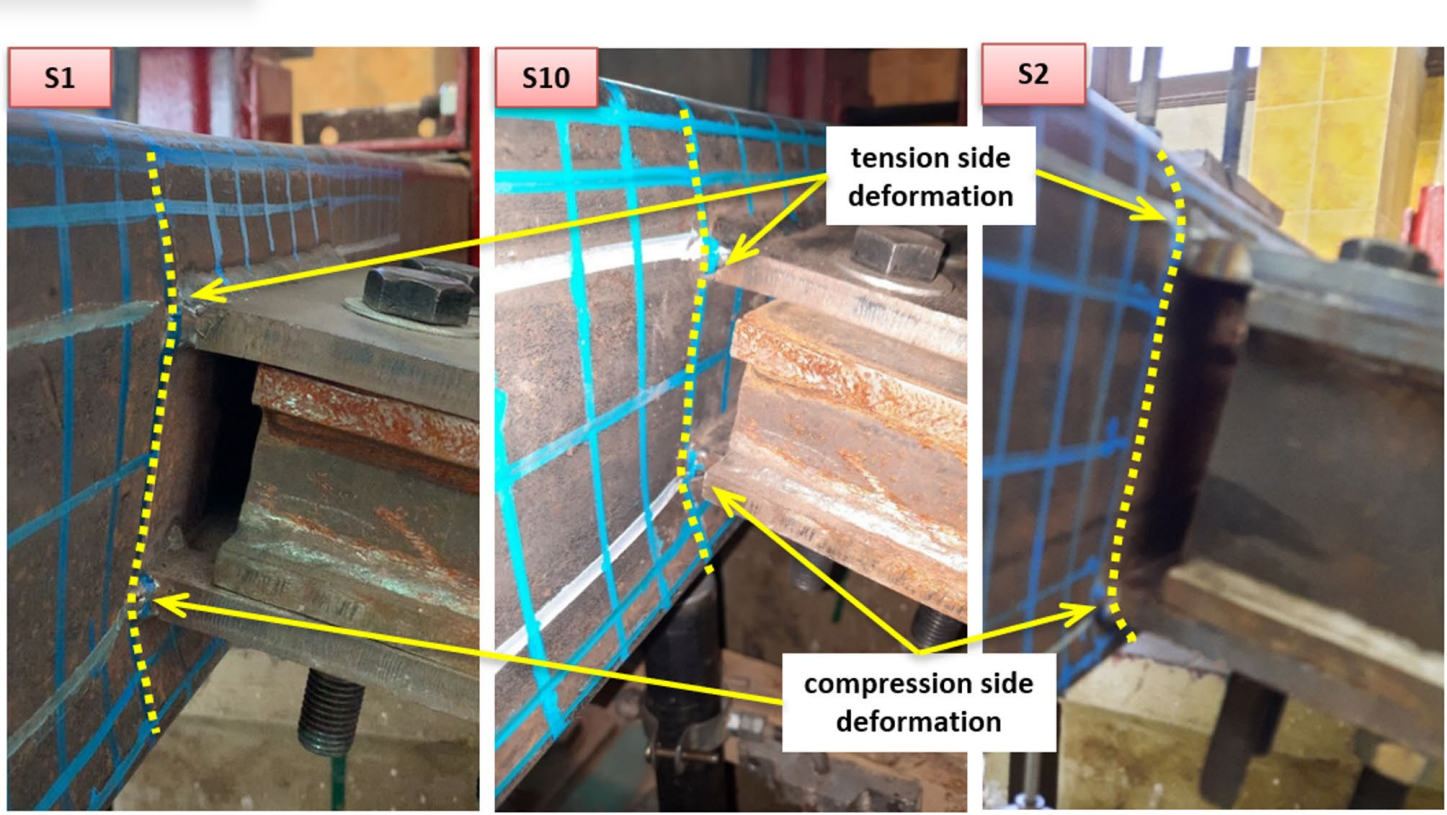
Failure modes of specimens S1, S2, and S10.

**Fig. 9:**
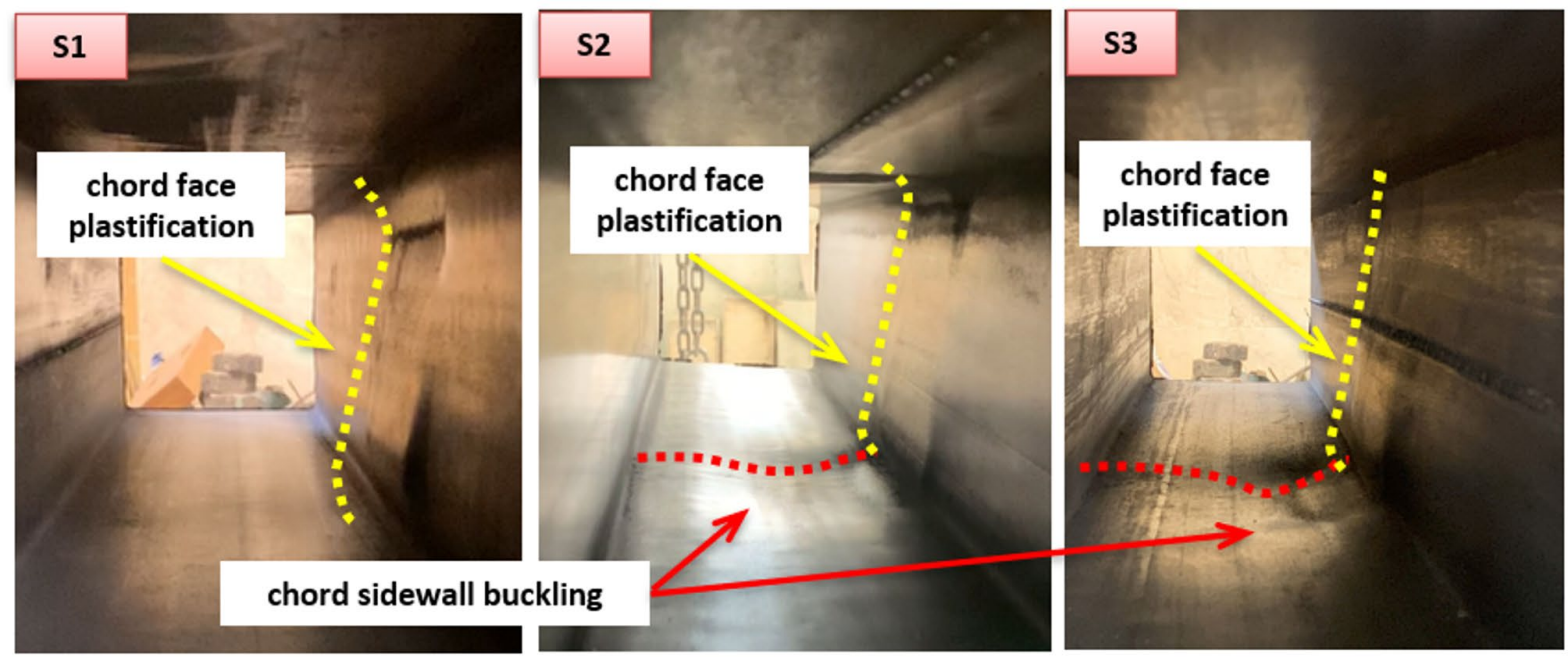
Failure modes of specimens S1, S2, and S3 (view from inside).

The failure mode of each specimen is presented in this section. In centric connection with a small β ratio, an anti-symmetric pattern of deformations in the chord face is observed at both plates. A sample of the failure mode is presented in Fig. 8 for specimens S1, S2, and S10. Inward and outward deformation at compression and tension zones, respectively, are observed with almost the same value. The specimens fail by pure chord face plastification. This is noticed in specimens S1 and S2 with β = 0.5 and 0.75, also S10 and S11 with the same configuration but having shorter welded plates (l_1_). This behavior is attributed to the plates being positioned further away from the chord sidewalls, resulting in a failure mechanism dominated by face bending. For S2, and with continuing the loading beyond the face plastification failure, sidewall buckling is observed at the bottom compression sidewall, as shown in Fig. 9. This is a result of getting the compression plate of the DLP closer to the chord sidewall, making the bottom sidewall subject to compression, so susceptible to local buckling. For specimens with a larger β ratio of 0.87 (S3 and S12), the deformations in the chord face at the two plates are no longer anti-symmetric. This different pattern is caused by the presence of the chord sidewall near the plate, which gives resistance against face deformation. Asymmetric deformation is detected due to the buckling in the bottom sidewall, which is located near the compression plate. Greater face deformation is observed at the bottom, which is not the case at the top, subject to tension. Local buckling in the bottom sidewall is observed.

**Fig. 10:**
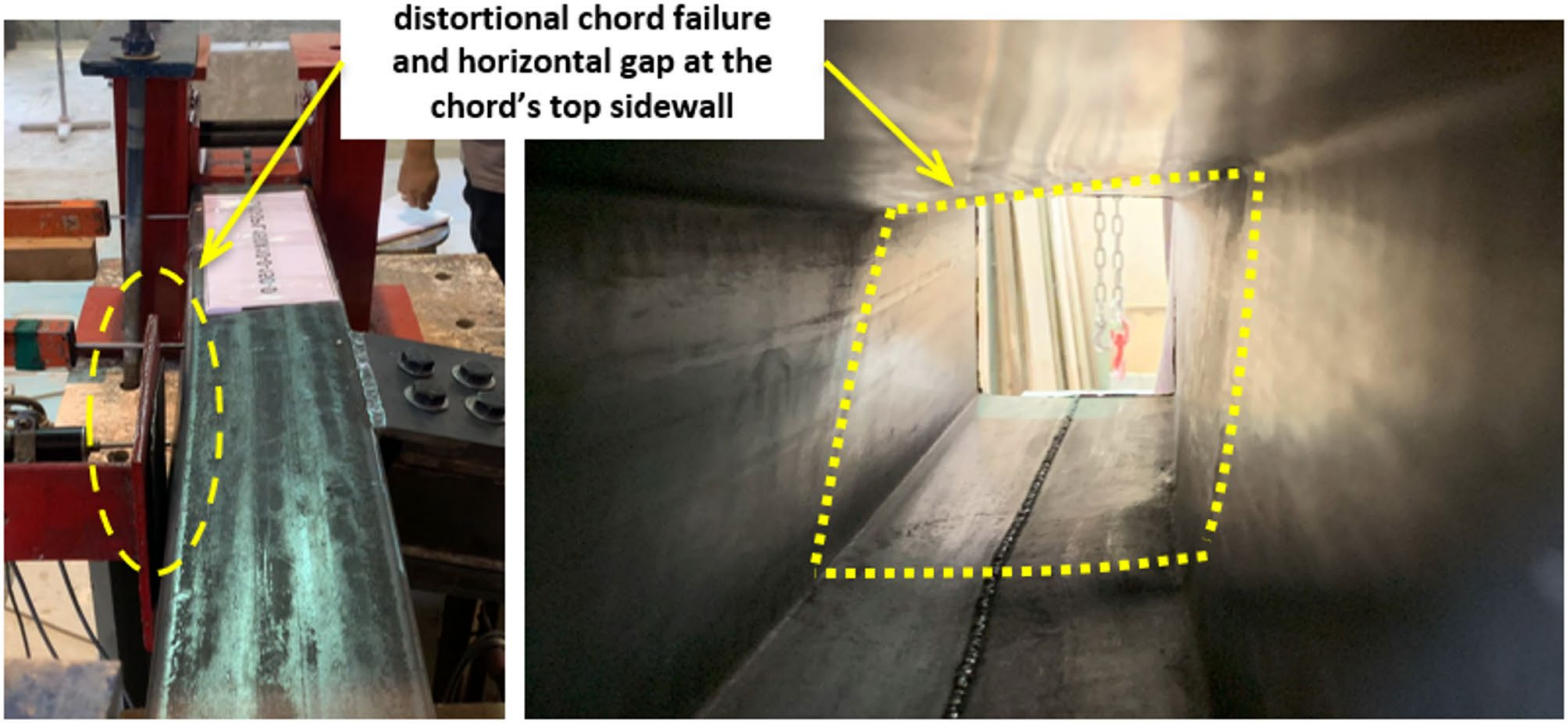
Failure mode of specimen S13.

For the specimen with β = 1.0 (S13), the double plates are welded directly to both sidewalls of the chord. As the configuration forms a T-joint, the connection is inherently unbalanced, making it more susceptible to chord distortion rather than sidewall buckling, as shown in Fig. 10. Although lateral bearing support is used during testing on the chord back face, this support prevents outward displacement of the bottom sidewall but cannot prevent displacement of the top sidewall away from the bearing support toward the load; thus, chord distortion ultimately governs the failure mode.

**Fig. 11:**
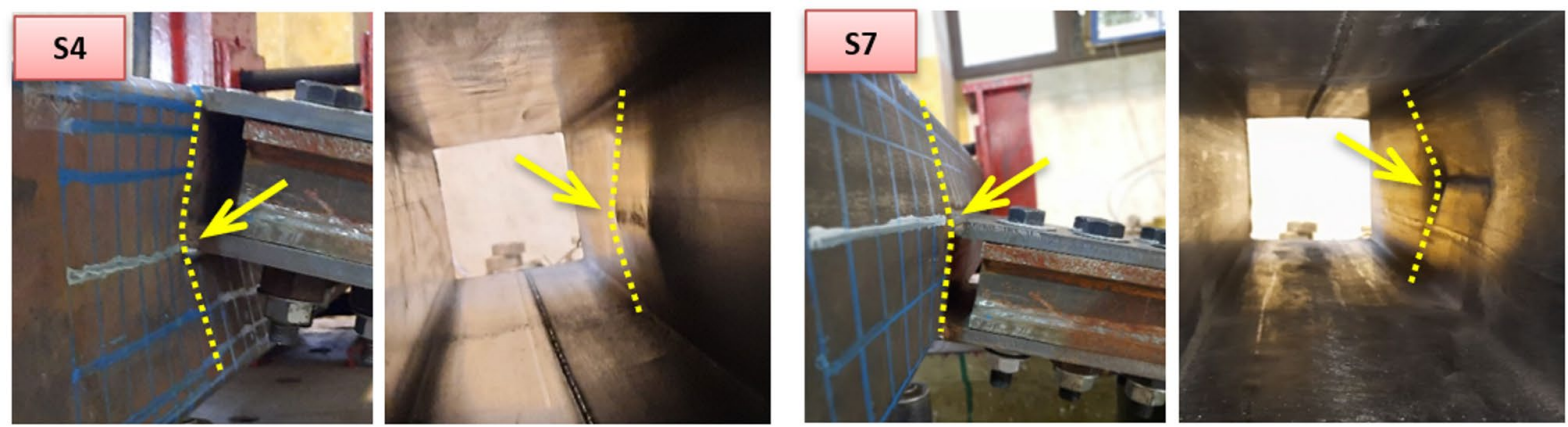
Failure modes of specimens S4 and S7.

Other tests are performed with eccentricity from the chord center line. Six eccentric DLP specimens are tested, three of which have top offset while the other three have bottom offset. No significant difference is observed in the failure mode of the two groups. Both configurations failed primarily due to chord face plastification, regardless the chord face is subject to inward compression force or outward tension force. For specimens with β = 0.5 (S4 and S7), as shown in Fig. 11, one of the double plates is welded to one chord sidewall while the other plate is welded exactly at the mid-depth of the chord face. Pure chord face plastification is the dominant failure mode. Despite the high stiffness of the sidewall in front of one plate, the other plate causes bending on the face, leading to chord plastification failure. This pattern is comparable to that of a single centric plate subjected to compression or tension.

**Fig. 12:**
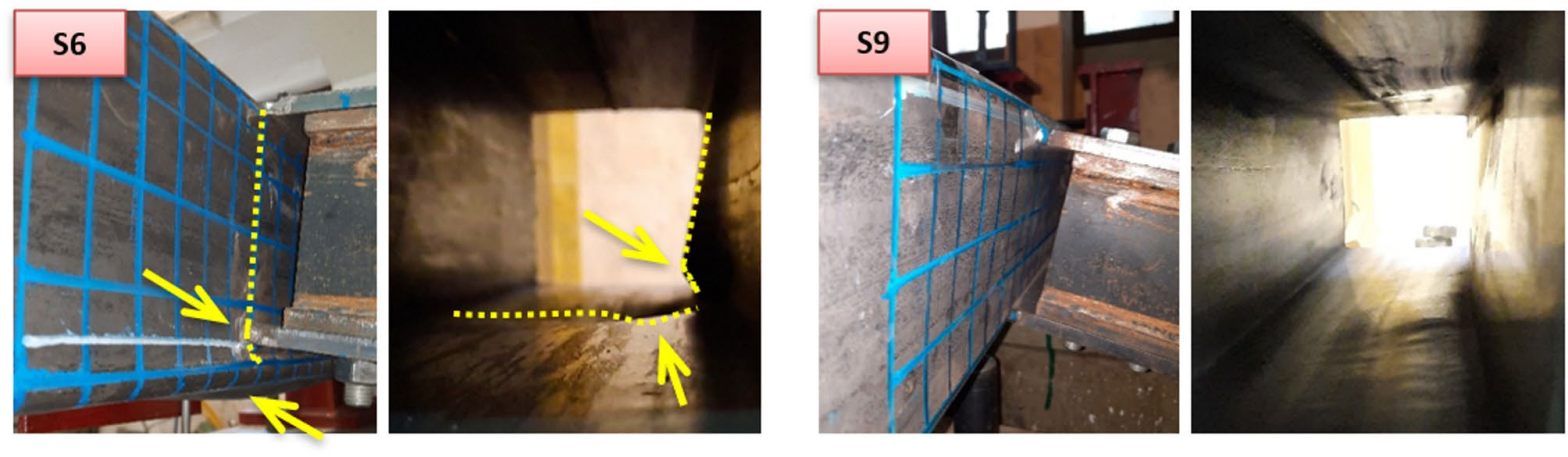
Failure modes of specimens S6 and S9.

For β = 0.87, a distinction is noted between the two offset cases. In the upward offset specimen S6—where the compression plate is located on the chord face and the tension plate on the sidewall—failure occurs through combined chord face plastification and sidewall buckling. Conversely, the downward offset specimen S9 promotes a failure mode dominated by pure chord face plastification, without the contribution of sidewall buckling. This can be observed in Fig. 12 for the failure mode of specimens S6 and S9. S6 with β = 0.875 and upward offset shows failure by chord face plastification combined with sidewall buckling.

### Load-deflection relationship

**Fig. 13:**
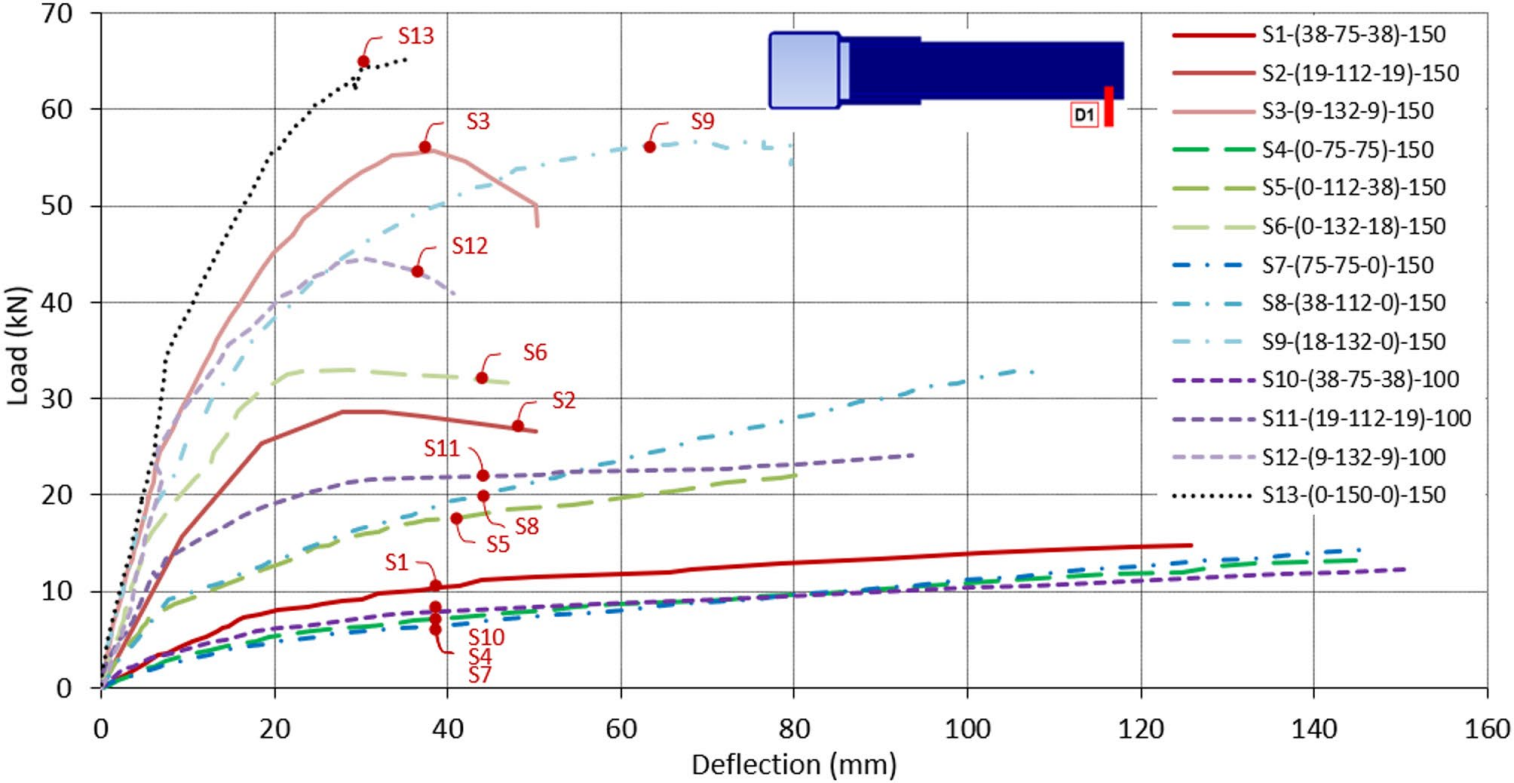
Load-deflection relationship for all specimens from LVDT D1.

The vertical deflection of the brace tip under the loaded point (D1) is measured during the test. The load-deflection relationships for all specimens are collected in the curves shown in Fig. 13. Referring to these different curves, all specimens exhibit elastic behavior at the beginning, transitioning to plastic behavior at the end. Some specimens reach their ultimate loads, followed by a decrease in load accompanied by an increase in deflection. However, the load in other specimens keeps increasing with no sign of a peak load. For the specimens where no peak load is detected and the loading continues to increase, the test is terminated when the stroke of the loading jack reaches its maximum value, reaching a deflection at the tip of the brace of around 150 mm.

It is observed that specimens with smaller β ratios, such as S1, S4, S7, and S10, tend to exhibit lower ultimate load capacity. These specimens continue to carry an increasing load without a distinct peak, indicating a gradual failure process. As the β ratio increases, specimens reach higher peak loads, as seen in S13, which has DLP spacing equal to the chord depth. In conclusion, the failure is primarily governed by the chord face. As long as the connection plates are positioned near the chord face center, excessive deformation is detected at low load. When the DLP spacing becomes wider and the double plates are positioned closer to the chord sidewall, a higher load is achieved due to the improved load transfers.

**Fig. 14:**
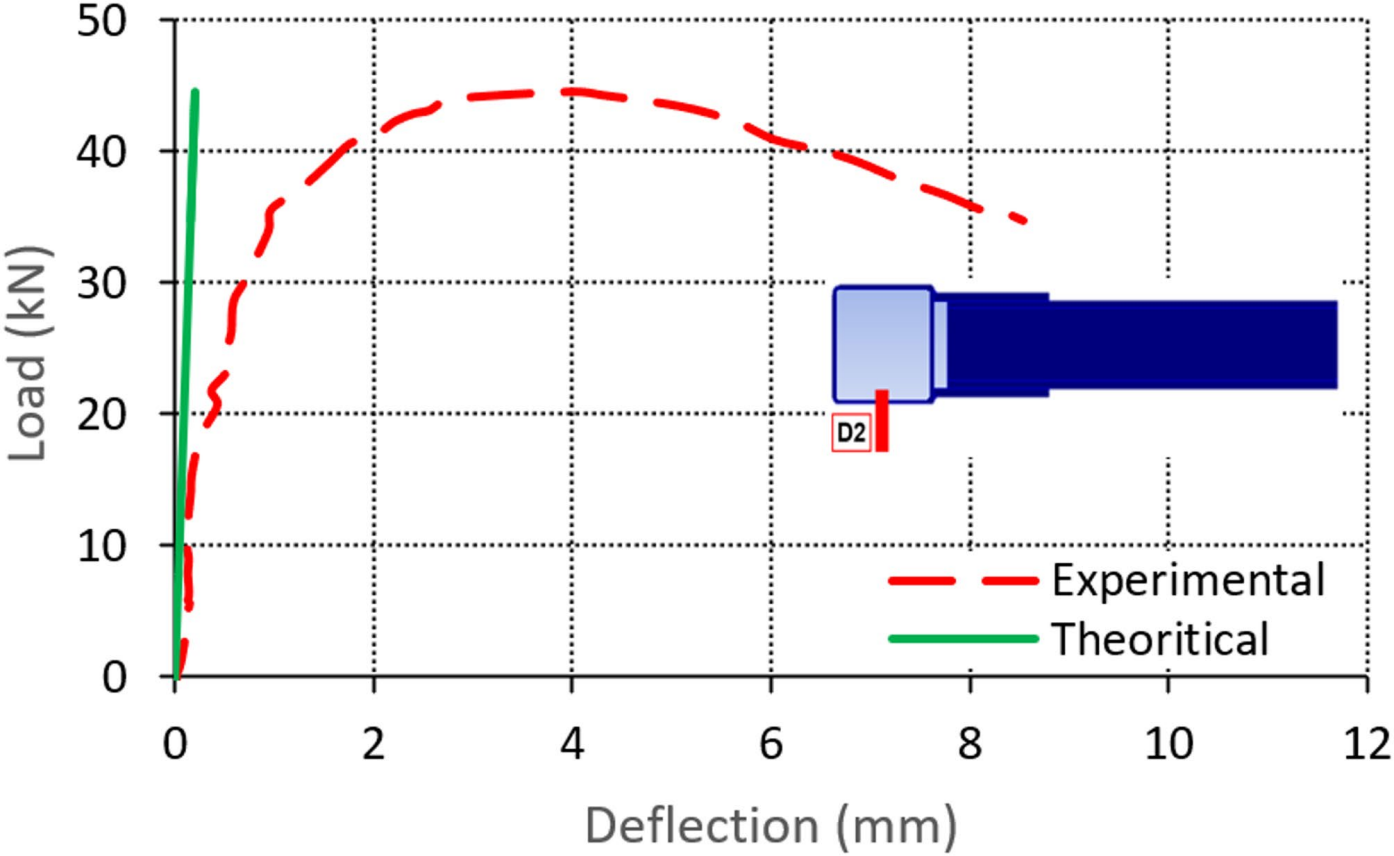
Load-deflection relationship for specimen S12 from LVDT D2.

The vertical deflection of the chord under its mid-span (D2) is measured during the test. In general, the deflection of the chord is found to be very small as a reason of the chord’s small span and the high second moment of inertia of the SHS section. However, a component of the measured deflection D2 comes from the local buckling of the chord’s bottom sidewall. A local buckling is detected at the bottom sidewall in many specimens due to the compression component from the moment. A sample for the load-deflection relationships for specimen S12 is presented in Fig. 14. It is noticed that the deflection at the beginning of the test has small values relative to the deflection calculated from the elastic theory. By increasing the load, local buckling starts at the bottom sidewall, and deflection values increase to high values deviated from the elastic theory of the beam. For reference, the elastic deflection of the chord is calculated as PL^3^/192EI, where P is the load, L is the span, E is the steel modulus of elasticity, and I is the second moment of inertia of the SHS member.

The top and bottom lateral displacements at the midspan of most of the specimens record minimal values. The displacement at the top sidewall D3 shows a tiny value not exceeding 0.5 mm in the majority of specimens. The displacement at the bottom sidewall D4 always tends to zero. Most of the specimens have no sign of distortion, except few specimens that show values, still low, of 1.0 to 1.5 mm horizontal deformation at the top sidewall measured by LVDT D3. The common factor in these specimens is the top plate, which is welded to the chord’s top sidewall in these cases. When the top plate of the DSLP is welded directly to the chord sidewall, the chord is more susceptible to distortion. For cases where the bottom plate is positioned in the chord face, like S4, the face plastification occurs first, averting the SHS distortion. However, as long as the bottom plate moves towards the bottom sidewall and away from the face mid-depth, face plastification fades and chord distortion appears. This is observed in S6. Generally, the distortion shown in the current tests cannot be defined as the main reason for failure, as the distorted specimens measure a maximum value of 1.5 mm difference between top and bottom sidewalls, which means only 1% sway of the SHS of 150 mm depth. However, this is not the case for S13, where the sway is more pronounced and the failure is mainly by section distortion. Based on the above, the rotation due to the twist of the HSS chord from the torsional moment at its midspan is considered minimal and ignored while calculating the rotations at the connection.

For the last LVDT, D5, which is positioned at the support, all the measured values are zero, evidencing the fixation of the support without any sign of sway or rotation.

### Moment-rotation behavior

**Fig. 15:**
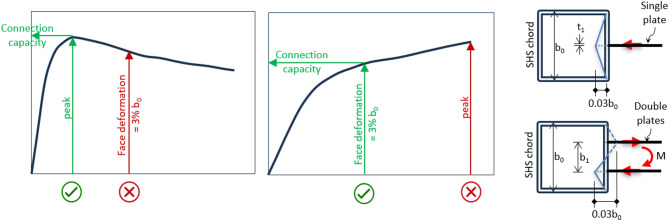
How to determine the connection capacity.

The load capacity of a single longitudinal plate to SHS connection subject to normal load on the chord face, either compression or tension, is well defined in previous research. This capacity can be calculated by considering the critical value from two ultimate limit states: (1) the peak ultimate load achieved, or (2) the load corresponding to deformation of 3%b_0_ in the chord face if no peak load is achieved^34^. This is illustrated in Fig. 15. For the case in this paper with a connection subject to an out-of-plane moment on the chord face, the deformation is a little different, but the same procedure is used. As a result of the moment, one side is deformed inward due to compression, while the other side is under tension and deformed outward. The moment resistance for the specimen is calculated when the deformation of the compression plus tension sides reaches 0.03b_0_, or reaches the peak load, whichever happens first.

**Fig. 16:**
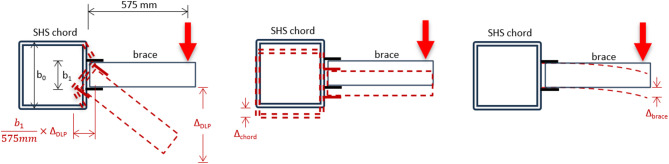
Determination of chord face deformation.

As illustrated before, the moment (M) in the experiment is calculated by multiplying the load by 575 mm, the distance from the applied load at the brace tip to the chord face where the DLP are welded. The connection capacity is obtained from the load-deflection relationship plotted for each specimen. To detect the deformation at the chord face, the rotation of the brace member is used, which is equal to the rotation of the chord face. Firstly, the vertical deflection at the brace tip is measured, then the joint rotation is calculated, and finally, the deformation of the chord face is derived. The total vertical deflection at the tip of the brace beam (Δ_D1_) is measured through the LVDT D1. This value involves three components as (Δ_DLP_), (Δ_brace_), and (Δ_chord_), as shown in Fig. 16. The vertical deflection Δ_DLP_ is the main unknown value that reflects the DLP connection rotation and can be used to calculate the horizontal deformation at the chord face. The vertical deflection Δ_brace_ is due to the vertical flexural deformation of the brace beam as a cantilever, so it is calculated theoretically by the elastic beam theory as a cantilever beam with a concentrated load at the beam tip. For reference, the elastic deflection of the cantilever brace is calculated as PL^3^/3EI, where P is the load, L is the cantilever span, E is the steel modulus of elasticity, and I is the second moment of inertia of the brace. The vertical deflection Δ_chord_ is due to the chord deflection and is calculated theoretically by the elastic beam theory as a fixed-fixed beam with a concentrated load at the beam midspan (illustrated in the previous sub-clause). The joint rotation is then calculated by dividing Δ_DSLP_ (which is equal to Δ_D1_ - Δ_brace_ - Δ_chord_) by 575 mm. The chord face deformation is derived by multiplying the joint rotation by b_1_, the spacing between the double plates of DLP. Using the above concept, the moment capacity of all specimens is calculated, and all results are summarized in Table 3. The table presents P_ult,_ which denotes the maximum load that occurs during the test, and presents its corresponding M_ult_. Also, M_Exp_ is presented in the table, which denotes the connection capacity that is calculated at the least from P_ult_ and the load that causes chord face deformation of 0.03b_0_.

**Table 3:** Results of experimental tests.

Specimen	*P*_ult_ (kN)	M_ult_ (kN m)	M_Exp_ (kN m)	Failure mode
S1	14.55	8.37	5.80	Chord face plastification at comp. & tens. plates. (Refer to Figs. 8 and 9)
S2	28.08	16.15	15.95	Chord face plastification at comp. & tens. plates, followed by sidewall buckling at comp. plate. (Refer to Figs. 8 and 9)
S3	55.72	32.04	27.05	Chord face plastification at comp. plate, accompanied by more pronounced sidewall buckling at comp. plate. (Refer to Fig. 9)
S4	13.22	7.6	3.86	Chord face plastification at comp. plate, accompanied by excessive brace inclination. (Refer to Fig. 11)
S5	23.24	13.36	8.05	Chord face plastification at comp. plate.
S6	31.91	18.35	18.19	Chord face plastification at comp. plate, followed by sidewall buckling at comp. plate. (Refer to Fig. 12)
S7	14.26	8.2	3.54	Chord face plastification at the tension plate, accompanied by excessive brace inclination. (Refer to Fig. 11)
S8	31.51	18.12	8.21	Chord face plastification at the tension plate.
S9	56.56	32.52	22.21	Chord face plastification at the tension plate, followed by little chord distortion. (Refer to Fig. 12)
S10	12.31	7.08	4.51	Chord face plastification at comp. & tens. plates, accompanied by excessive brace inclination. (Refer to Fig. 8)
S11	21.28	12.24	11.59	Chord face plastification at comp. & tens. plates, followed by sidewall buckling at comp. plate.
S12	43.83	25.2	23.02	Chord sidewall buckling at comp. plate.
S13	65.21	37.5	31.88	Chord distortion. (Refer to Fig. 10)

## Effects of key parameters on connection behavior

The connection moment capacity and the failure mode of each specimen are summarized in the last section in Table 3. Generally, the observed failure mode of the centric DLP connection is mainly chord face deformation. Buckling is detected at the chord’s bottom sidewall, which is located at the compression zone of the bending moment. At the chord’s top sidewall, which is located at the tension zone of the moment, no tearing or elongation is detected, but chord distortion, either occurring individually or in combination, is detected in some models. The brace inclines to its original horizontal position, but with no indication of local failure or bending of the brace member.

In the next sections, a discussion on the effect of different parameters is presented, including (1) double plate spacing -to- chord depth ratio, (2) plate width -to- chord depth ratio, (3) the offset of the connection, and (4) stiffness and ductility.

### Effect of double plate spacing-to-chord face depth ratio (β)

**Fig. 17:**
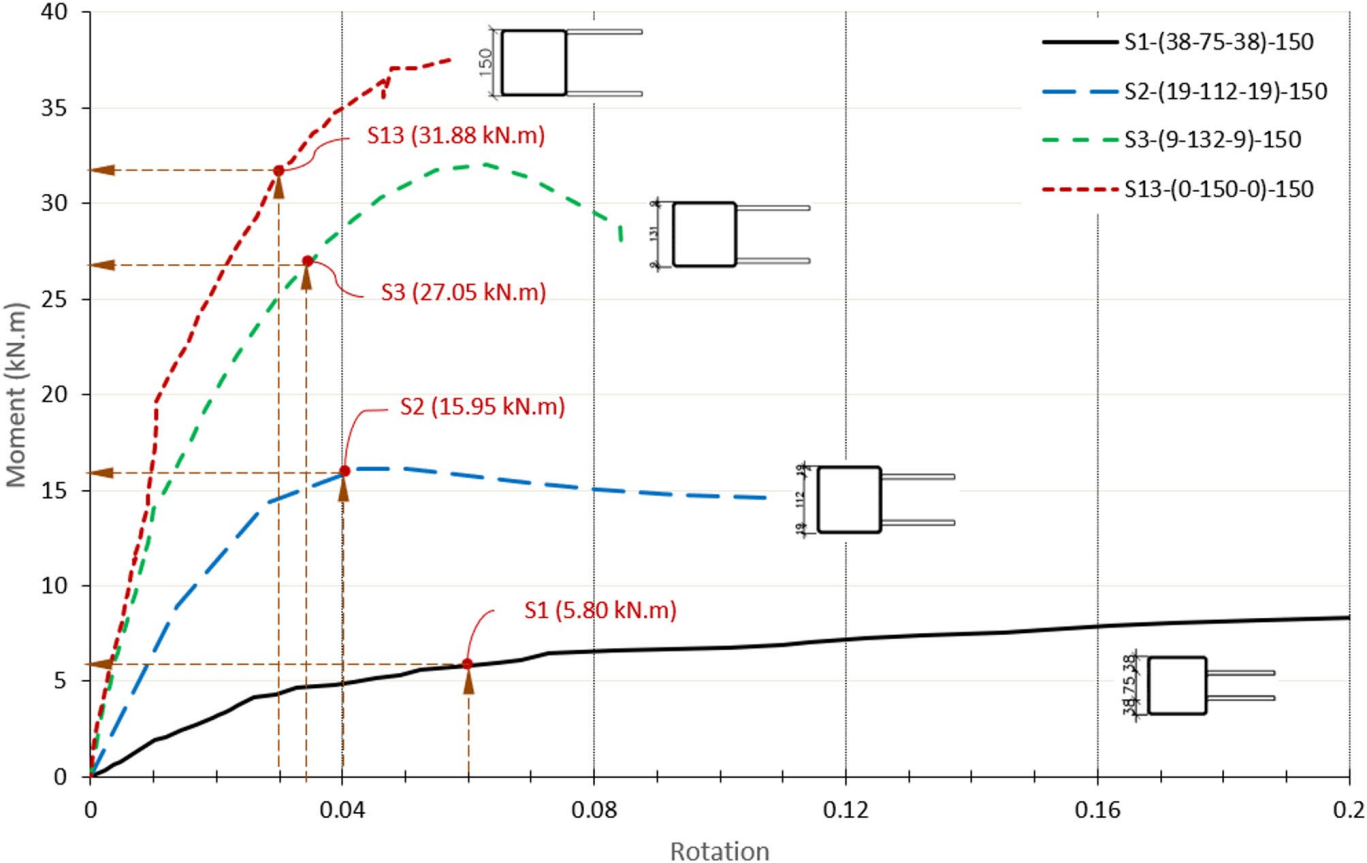
Moment-rotation relationship of centric DLP connection for ɳ = 1.0.

The failure patterns are influenced by the β ratio of the specimens and the positioning of the double plates to the chord sidewalls. As shown in Fig. 17, the moment capacity M_Exp_ increases with larger double plate spacing in centric configurations. Contrary, the ductility and the rotation decrease with increasing β ratio. All these capacities occur at face deformation of 0.03b_0_, while the load keeps increasing after that to its peak point. The corresponding rotations are 0.06, 0.04, 0.034, and 0.03 for β ratios of 0.5, 0.75, 0.875, and 1.0, respectively. For β = 0.5, the connection capacity M_Exp_ is equal to 5.8 kN m. This capacity significantly increases to 15.95 kN m and 27.05 kN m for β = 0.75 and 0.875, respectively. The increase in the connection capacity continues as β becomes close to unity. The capacity of β = 0.75 is 2.75 times that of β = 0.5. The enhancement continues, and the capacity of β = 0.875 reaches 1.7 times that of β = 0.75. This trend reflects a transition in the failure mechanisms from chord face plastification, which governs the response, with no significant sidewall deformation observed within the deformation limit. As β increases and the plates are positioned closer to the chord sidewalls, the failure mode progressively shifts toward sidewall buckling, which provides enhanced stiffness and improved load resistance.

For the specimen with β = 1.0 (S13), the double plates are welded directly to both sidewalls of the chord, resulting in the highest moment resistance among all tested specimens, equal to 31.88 kN m (corresponding to 3% b_0_ deformation) and an ultimate moment of 37.5 kN m as shown in Fig. 17. The capacity increases 650%, from 5.8 kN m for β = 0.5 to 37.5 kN m for β = 1.0. This superior capacity is primarily due to the increased connection stiffness provided by direct welding, which enhances force transfer and omits local deformation and bending in the chord face.

### Effect of double plate width -to- chord face depth ratio (ɳ)

**Fig. 18:**
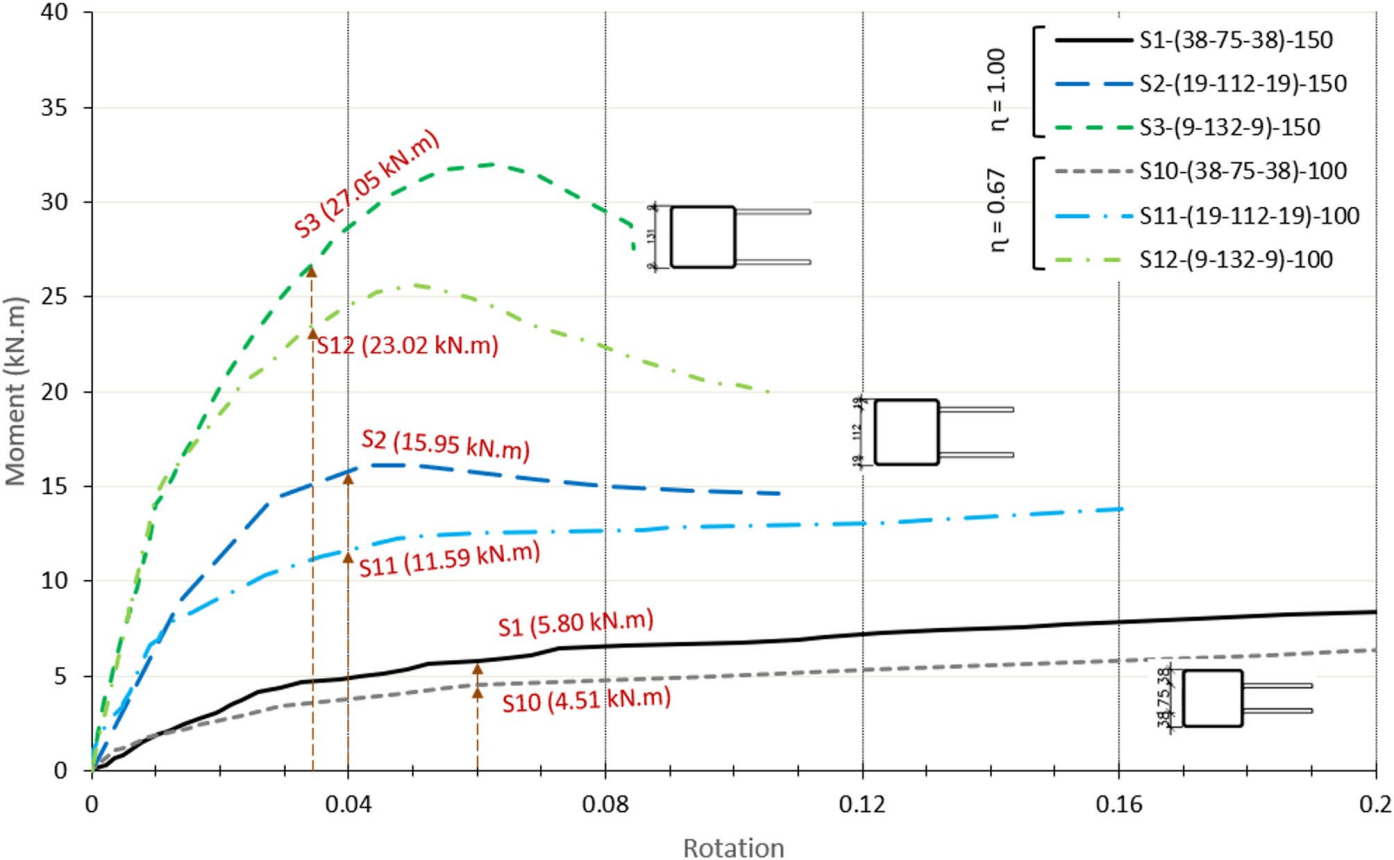
Moment-rotation relationship of centric DLP connection for different ɳ ratios.

The connection capacity is influenced by the η ratio of the specimens, which reflects the width of DLP to the depth of the chord. Decreasing η leads to a deterioration in the moment capacity of the connection. As shown in Fig. 18 for the moment-rotation relationship of specimens with η = 0.67, the same trend with β is noticed, and an increase in the connection capacity continues as β becomes close to unity. The capacity of β = 0.75 is 2.57 times that of β = 0.5 (11.59 to 4.51 kN m). The enhancement continues, and the capacity of β = 0.875 reaches 1.99 times that of β = 0.75 (23.02 to 11.59 kN m).

**Fig. 19:**
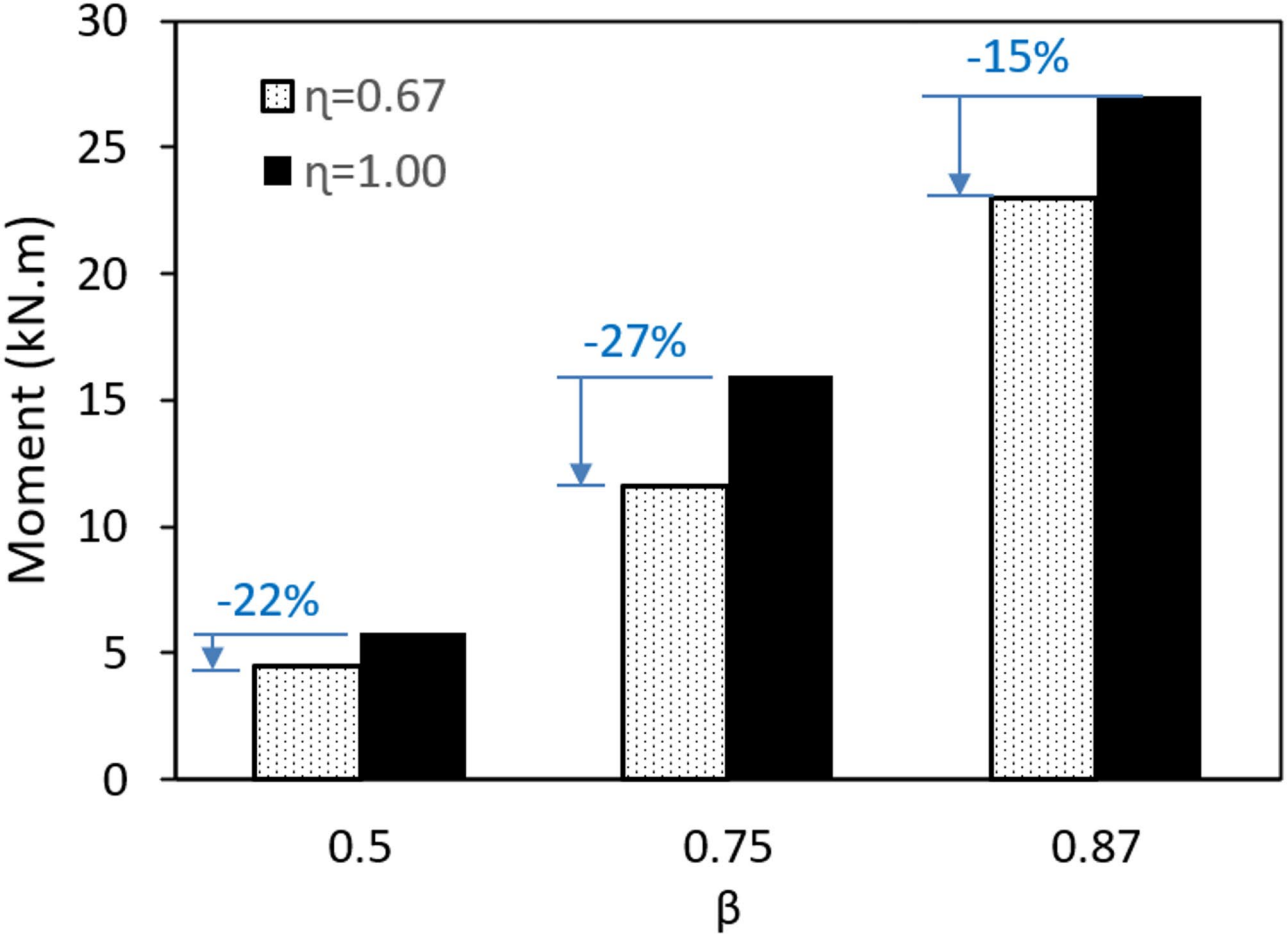
Comparison between ɳ = 1.0 and 0.67.

Comparing the results of different plate widths, the moment capacity of all specimens with l_1_ = 100 mm ( η = 0.67) is found to be lower than the same sections with l_1_ = 150 mm ( η = 1.0). The ratios are presented in Fig. 19 using a column chart, showing a decreasing ratio in the range of 15–27%. Specimens with the same β ratio exhibit identical failure modes. For β = 0.50 and 0.75, where the governing failure is chord face plastification, decreasing η from 1.00 to 0.67 results in an average of a 25% decrease in the moment capacity. In contrast, for β = 0.87, where the failure involves a combination of chord face plastification and sidewall buckling, the decrease in the capacity is a little lower, reaching 15%. This difference can be attributed to the influence of the more complex failure mechanism at higher β ratios, including sidewall buckling. Reducing the plate width concentrates the force on the chord face, making the face weaker against the wall plastification. However, this is less shown in high β ratios when sidewall buckling gets involved, which is more sensitive to the wall thickness and width rather than the plate width.

Connecting the brace to the chord through a plate, instead of welding the brace directly to the chord, offers a significant advantage for enhancing the connection capacity. While the brace itself may have a relatively small width, utilizing a wider plate at the connection can improve load distribution, reduce localized stresses, and increase connection resistance as concluded. This approach allows the connection to engage a larger portion of the chord face, effectively promoting more uniform force transfer. Furthermore, the length of the connection along the brace axis plays a crucial role in ensuring adequate load transfer with minimum shear lag effect. According to AISC Design Guide 24 (Second Edition, 2024)^35^ and AISC 360 − 22^20^, a minimum slope of 1:2.5 (i.e., horizontal length ≥ 2.5 × plate thickness) is recommended for gusset plates to provide effective force dispersion and limit stress concentrations. Therefore, adopting a wider and properly sloped plate not only aligns with code requirements but also enhances the connection capacity of DLP connections.

### Effect of DLP eccentricity

**Fig. 20:**
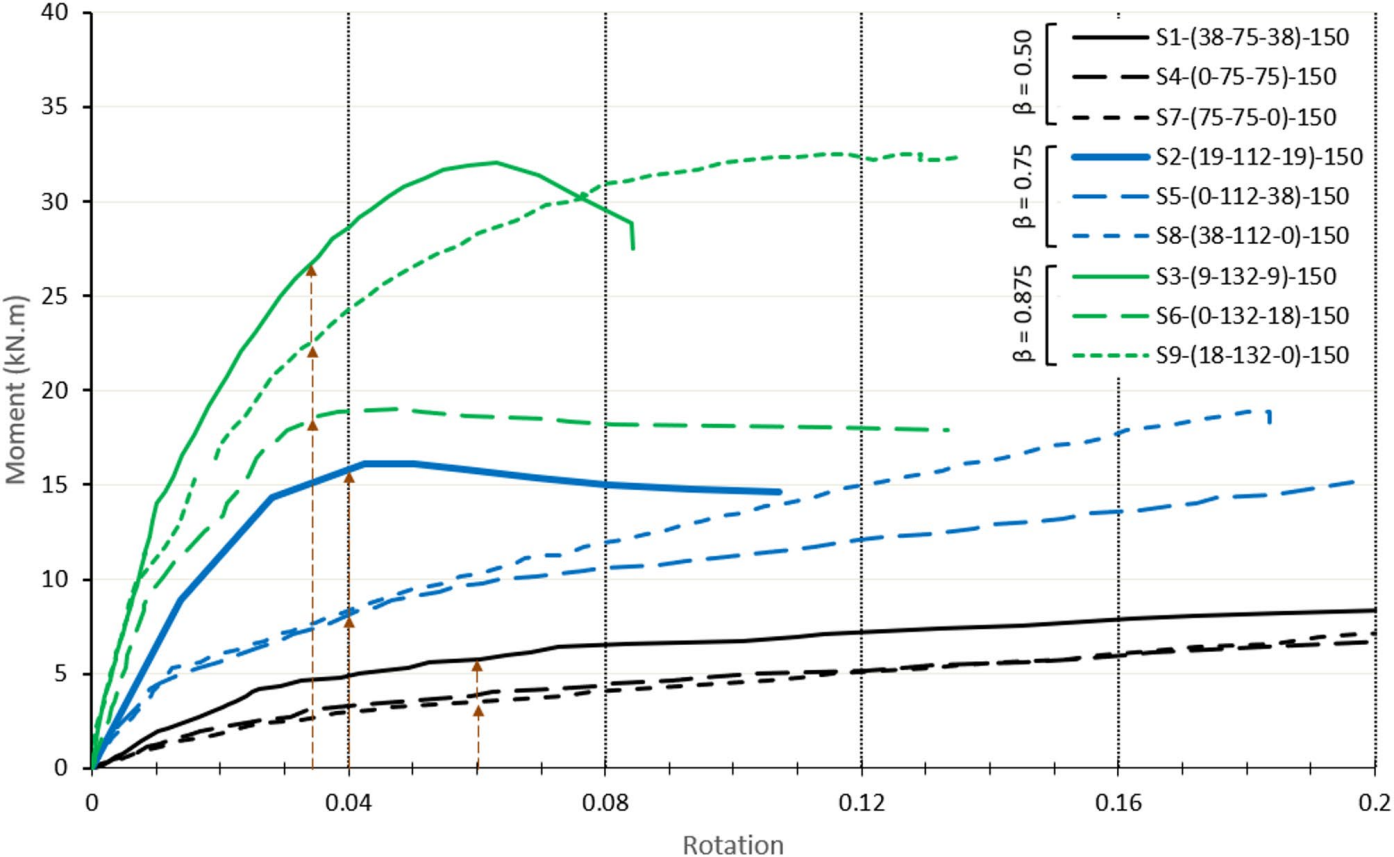
Moment-rotation relationship of eccentric DLP connection for different β ratios.

A total of six eccentric DLP specimens are compared with three centric specimens at equivalent β ratios. As shown in Fig. 20 for the moment-rotation relationship of specimens with different β ratios, the results indicate that centric connections (S1, S2, and S3) exhibit higher moment capacities than their eccentric counterparts for the same β ratio. This improvement in strength can be attributed to the location of the double plates in the centric configuration, which are positioned equal distance around the chord face mid-depth so closer to the sidewalls. However, for the same β ratio, one of the double plates in the eccentric connections is positioned at one sidewall, while the other plate is positioned near the face mid-depth and far from the sidewall. This concentrates the load in the most elastic point of the chord face.

In examining the effect of top and bottom offset configurations in eccentric DLP connections, as shown in Fig. 20, no significant difference in the capacity is observed for β = 0.5 and 0.75. For specimens with β = 0.5 (S4 and S7), where one of the double plates is welded at the mid-depth of the chord face, the lowest moment resistance among all tested specimens is reached. Specimens with β = 0.75 (S5 and S8) also exhibited a pure chord face plastification failure mode rather than buckling of the chord sidewall, but with higher moment resistance after moving the plate on the chord face away from the mid-depth. For β = 0.87, the upward offset specimen S6, with the tension plate on the sidewall, shows a lower capacity than the downward offset specimen S9 with the compression plate on the sidewall. The tension plate, being located on the chord face in S9, promotes a pure chord face plastification failure mode without any sidewall buckling.

## Relative design rules

The current design codes and guidelines, such as those outlined in AISC Design Guide No. 24^35^ and others^19–22^, provide provisions for connections to the SHS chord. The two configurations that are found to be the most relevant to the study in this paper are the single longitudinal plate subject to normal force of tension or compression, and the SHS-to-SHS connections subject to out-of-plane moment. The case with a single plate is similar to the eccentric DLP, where one plate is positioned at a sidewall while the other is positioned at the center of the chord face (S4 and S7). The other case with SHS-to-SHS is similar to the concentric DLP, where the double plates are positioned at equal distances around the center of the chord face, ignoring the two webs existing in the SHS brace (S1 to S3, and S10 to S12).

### Case of centric connection with β < 1.0

Starting with the available design rules for SHS-to-SHS connections subject to out-of-plane moment, one of several failure modes may govern these connections. These failure modes include chord face plastification, local brace failure, shear punching, chord distortion, and sidewall buckling or yielding. In the current study, the dimensions of the connection and brace are chosen stiff enough to avoid any local failure on them, thus limiting the failure modes to be in the chord only, with face plastification or sidewall buckling only. Local buckling in the brace compression flange is avoided by considering a thick plate with a slenderness ratio away from the slender element limit. Lateral torsional buckling of the brace is avoided by using a closed section (tube) instead of an open section (I-shape). Brace yielding is evaluated based on the plastic moment capacity of the brace section, to be much higher than the connection capacity. Chord distortion is minimized by providing lateral support at the chord mid-span on the lower sidewall. Shear punching is assessed based on the limits defined in AISC 360 − 22^20^, and the chord of 4.5 mm and plates of 100 and 150 mm satisfy the specified criteria. None of these failure mechanisms (brace yielding, chord distortion, or shear punching) is observed during testing as planned.

**Fig. 21:**
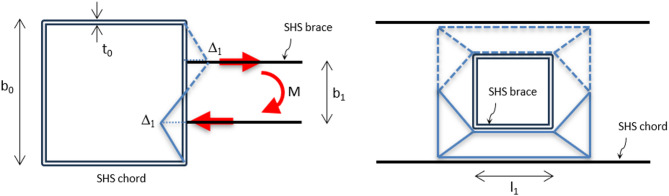
Yield line theory of SHS-to-SHS connection.

As a demarcation line in the code, the failure of connections with β ≤ 0.85 is predominantly characterized by chord face plastification, while it is sidewall buckling for β > 0.85. The moment capacity of this connection, considering chord face plastification, is calculated using the following associated design equation. This equation is based on the yield line mechanism as illustrated in Fig. 21.

1 $$\:{M}_{code1}={F}_{y}{{t}_{0}}^{2}\:\left[\frac{{l}_{1}\left(1+\beta\:\right)}{2\left(1-\beta\:\right)}+\sqrt{\frac{2\:{b}_{0}\:{b}_{1}\:\left(1+\beta\:\right)}{\left(1-\beta\:\right)}}\right]$$

Where F_y_ = yield strength, t_0_ = chord thickness, l_1_ = brace width (presented as the plate width of the DLP in this paper), β = brace depth -to- chord face depth ratio (b_1_/b_0_), b_1_ = brace depth (presented as the out-to-out distance of the double plates in this paper), b_0_ = chord face depth.

A comparative analysis is conducted between the experimentally measured moment capacities of the concentric DLP specimens and the values predicted by the available code as given in Eq. 1, AISC Design Guide No. 24^35^. As summarized in Table 4, the results indicate that the current provisions are overly conservative, consistently underestimating the true resistance of DLP connections. Furthermore, the difference between the experimental and theoretical values becomes more pronounced for a high β ratio. The ratios are 1.48 and 1.36 for β = 0.5. However, the ratios are in the range of 1.74 to 1.96 for β = 0.75 and 0.875.

### Case of eccentric connection with β = 0.5 and a plate at the chord face mid-depth

**Fig. 22:**
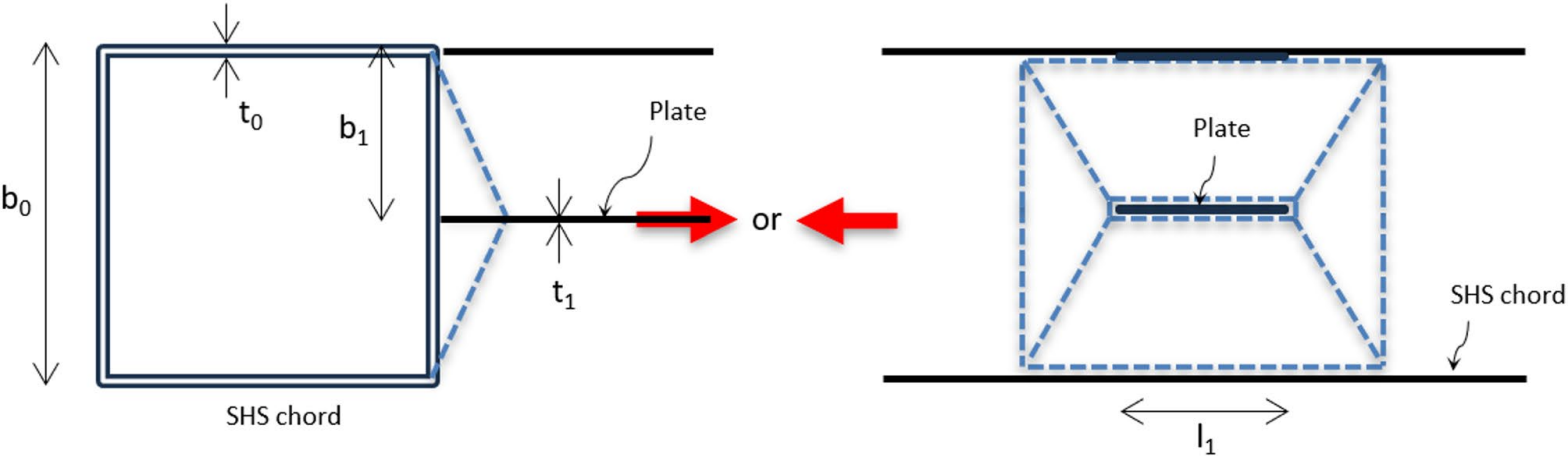
Yield line theory of centric single longitudinal plate connection.

Further evaluation is performed on the second relative case available in the design codes using the design rules for a single longitudinal plate connection subject to normal force. The code provisions primarily address the scenario of a centric plate, assuming a pure chord plastification failure mode. The load capacity of this connection (P_singlePL_), considering chord face plastification, is calculated using the following associated design equation, Eq. 2, AISC Design Guide No. 24^35^. This equation is based on the yield line mechanism as illustrated in Fig. 22. For comparison purposes, P_singlePL_ is multiplied by the outer spacing between the double plates (b_1_) – neglecting the thickness, similar to SHS-to-SHS rules where thickness is not considered – to estimate the moment capacity of an eccentric DLP connection (M_code2_), as given in Eq. 3. This approach enables a direct evaluation of eccentric DLP with β = 0.5, where one plate is positioned at the chord sidewall and the other at the face mid-depth (S4 and S7).

2 $$\:{P}_{singlePL}=\:{F}_{y}\:{{t}_{0}}^{2}\:\left[\frac{{2l}_{1}/{b}_{0}}{1-\:{t}_{1}/{b}_{0}}+\frac{4}{\sqrt{1-{t}_{1}/{b}_{0}}}\right]$$

3 $$\:{M}_{code2}={P}_{singlePL}\times\:{b}_{1}\:$$

Where t_1_ = plate thickness.

**Fig. 23:**
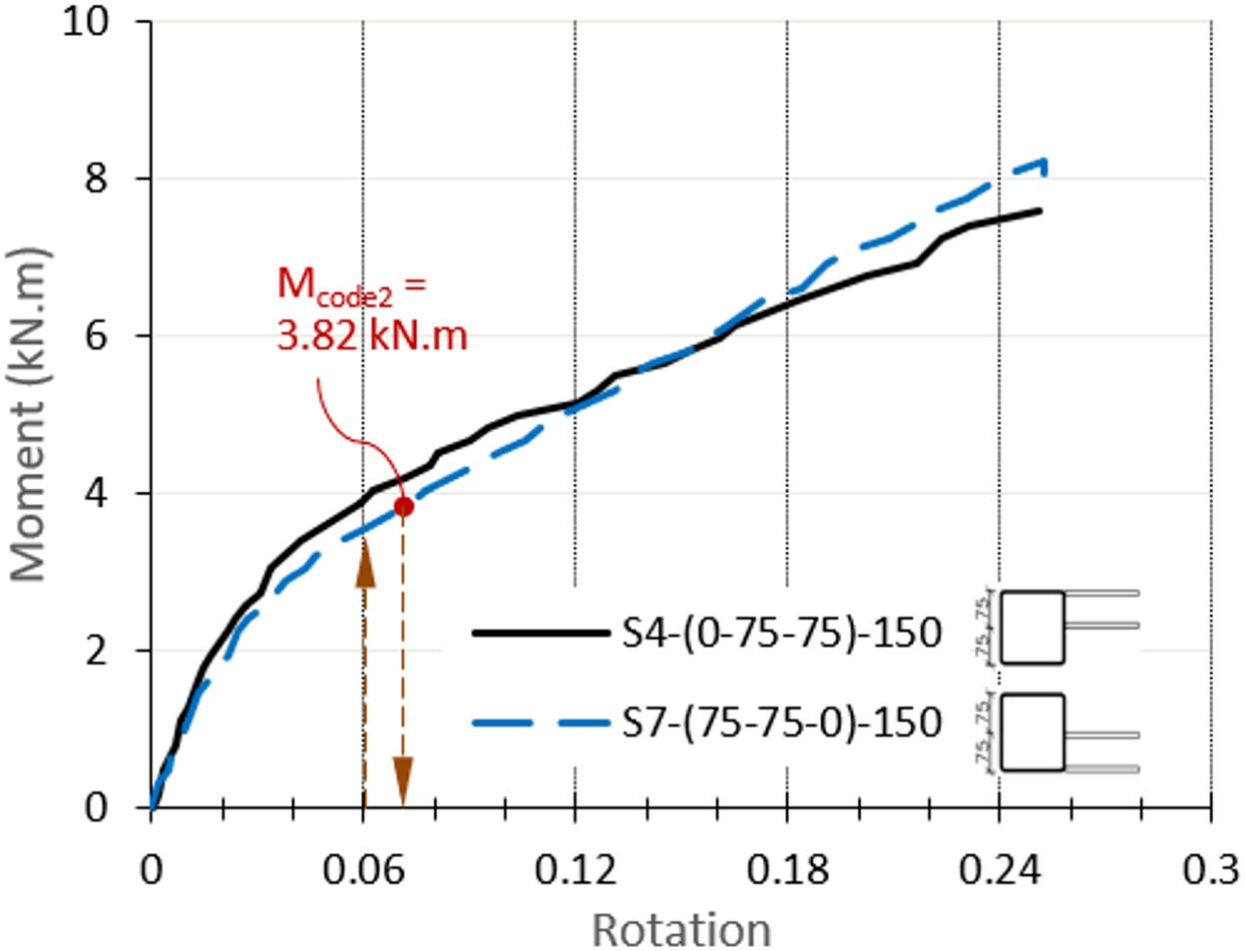
Moment-rotation relationship of eccentric DLP connection for S4 and S7.

As shown in Table 4, this method yields results that are close to the experimental results. S4 shows a ratio equal to 1.01, while S7 is on the unconservative side with a ratio of 0.93. Reference to Fig. 23, which presents the moment-rotation relationship of S4 and S7, the moment keeps increasing after the moment capacity corresponding to the 3% b_0_ deformation, and the ultimate load reaches double the proposed capacity. From the curves, it can be noticed that the experimental moment is equal to M_code2_ at a rotation of 0.07. This rotation corresponds to face deformation of 0.035b_0,_ very close to the proposed limit of 0.03b_0_, so is considered as a minor and acceptable shifting Accordingly, it can be assumed that the design code for a single plate subject to normal force is still applicable and can be used and multiplied by 0.5b_0_ to calculate the moment capacity of an eccentric DLP connection with β = 0.5.

### Case of eccentric connection with β > 0.5 and a plate away from the chord face mid-depth

**Fig. 24:**
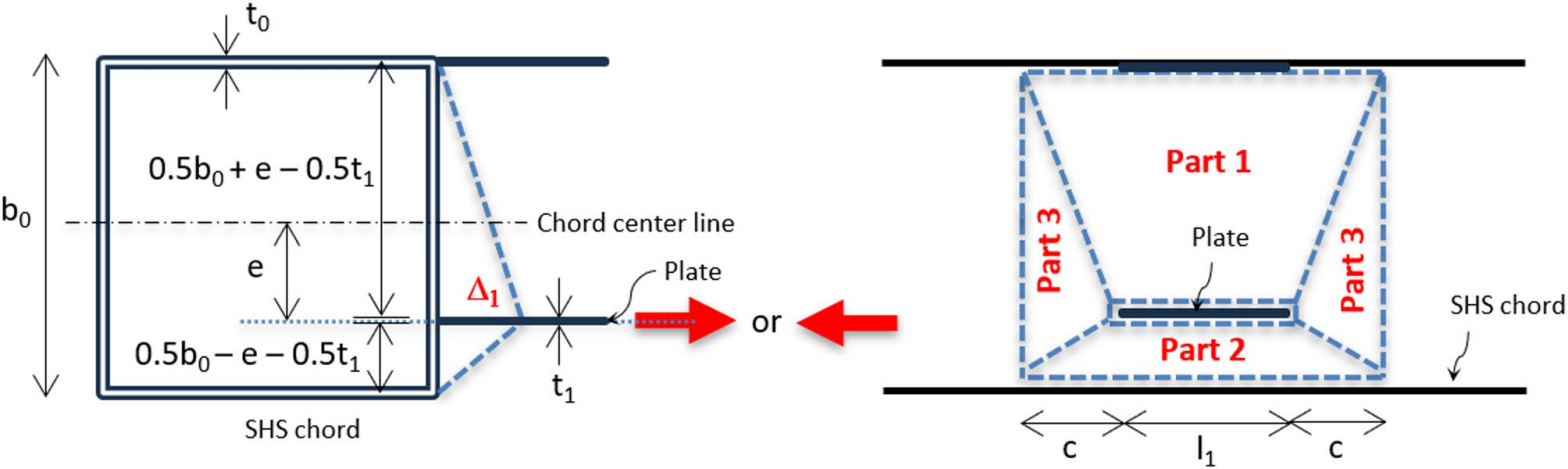
Yield line theory of eccentric single longitudinal plate connection.

Due to the higher stiffness of the chord sidewall, the critical plate in the eccentric configurations is the one welded to the chord face, which can either be aligned with the chord face mid-depth, as discussed in the last paragraph, or shifted from the face center, depending on the β ratio. To address the identified limitations in the stress distribution analysis of the shifted plate, a modified yield line model from that of a single plate at the center is proposed, as shown in Fig. 24. This model incorporates the effect of the plate’s offset from the longitudinal centerline of the SHS chord. A new parameter (e) is introduced to define the shift of the single plate from the chord’s centerline. The proposed yield line pattern accounts for the asymmetric stress distribution. By integrating the eccentricity term (e) into the yield line formulation, the revised model aims to improve the prediction accuracy of the moment resistance in eccentric DLP configurations, ensuring better alignment with experimental findings. As shown in Table 4, the proposed equation gives good agreement with S5 and S8 with ratios of 1.19 and 1.22, while showing conservative results for S6 and S9.

Based on the principle of energy conservation, the external work exerted by the axial load P_singlePLecc_ is equal to the total internal plastic energy dissipated along the yield lines (Parts 1 to 3), as illustrated in Fig. 24.

4 $$\:{P}_{singlePlecc}\:{\varDelta\:}_{1}=\:\sum\:_{i=1}^{3}{E}_{i}\:$$

Each internal energy component is expressed as $\:{E}_{i}=n\mathcal{l}\varphi\:{m}_{p}$, where n is the number of yield lines in each segment, $\:\mathcal{l}$ and ϕ represents the length and rotation of the yield line, respectively, and m_p_ is the plastic moment per unit length of the chord wall (m_p_ = F_y_t_o_^2^ /4). The energies of the three yield line segments in Fig. 24 are:

5a $$\:{E}_{1}=2\left({l}_{1}+2c\right)\left(\frac{{{\Delta\:}}_{1}}{0.5{b}_{0}-e-0.5{t}_{1})}\right){m}_{p}$$

5b $$\:{E}_{2}=2\left({l}_{1}+2c\right)\left(\frac{{{\Delta\:}}_{1}}{0.5{b}_{0}+e-0.5{t}_{1}}\right){m}_{p}$$

5c $$\:{E}_{3}=4\left({b}_{0}\right)\left(\frac{{{\Delta\:}}_{1}}{c}\right){m}_{p}$$

By substituting these expressions into the energy balance equation, the term ∆_1_ cancels out, leaving an equation dependent on the unknown parameter c. Minimizing the total energy with respect to c leads to:

6 $$\:c=\sqrt{\frac{{b}_{0}(0.5{b}_{0}-e-0.5{t}_{1})(0.5{b}_{0}+e-0.5{t}_{1})}{({b}_{0}-{t}_{1})}}$$

Substituting this result back into the equilibrium expression gives the final form of the axial load capacity for the eccentric single plate configuration:

7 $$\begin{aligned}\:{P}_{singlePLecc} & = {F}_{y}\:{{t}_{0}}^{2}\:\left[\frac{{l}_{1}\left({b}_{0}-{t}_{1}\right)}{2\left(0.5{b}_{0}-e-0.5{t}_{1}\right)\left(0.5{b}_{0}+e-0.5{t}_{1}\right)}+2\sqrt{\frac{{b}_{0}\left({b}_{0}-{t}_{1}\right)}{\left(0.5{b}_{0}-e-0.5{t}_{1}\right)\left(0.5{b}_{0}+e-{0.5t}_{1}\right)}}\right]\\ & =\:{F}_{y}\:{{t}_{0}}^{2}\:\left[\frac{{2l}_{1}/{b}_{0}}{\left(1-\frac{{t}_{1}}{{b}_{0}}\right)-\frac{{4e}^{2}/{{b}_{0}}^{2}}{\left(1-\frac{{t}_{1}}{{b}_{0}}\right)}}+\frac{4}{\sqrt{(1-\frac{{t}_{1}}{{b}_{0}})-\frac{{4e}^{2}/{{b}_{0}}^{2}}{\left(1-\frac{{t}_{1}}{{b}_{0}}\right)}}}\right]\end{aligned}$$

8 $$\:{M}_{proposed}={P}_{singlePLecc}\times\:{b}_{1}\:$$

This equation, Eq. 7, tends to Eq. 2 when e = 0 for the case of the plate exactly at the chord center line. Also, the eccentricity (e) can be applied in the equation in + ve or -ve sign, giving the same capacity of the normal force, but different moment capacity based on b_1_ value in Eq. 8.

**Table 4:** Comparison between experimental results and relative design rules.

Specimen	M_Exp_ (kN m)	M_code1_ (kN m)	M_code2_ (kN m)	M_proposed_ (kN m)	M_Exp_/ M_code_
S1	5.80	3.93	--	--	1.48
S2	15.95	8.14	--	--	1.96
S3	27.05	15.16	--	--	1.78
S4	3.86	--	3.82	--	1.01
S5	8.05	--	--	6.72	1.19
S6	18.19	--	--	11.34	1.61
S7	3.54	--	3.82	--	0.93
S8	8.21	--	--	6.72	1.22
S9	22.21	--	--	11.34	1.95
S10	4.51	3.32	--	--	1.36
S11	11.59	6.68	--	--	1.74
S12	23.02	12.17	--	--	1.89
S13	31.88	--	--	--	--

### Proposal to calculate the DLP connection moment capacity

**Fig. 25:**
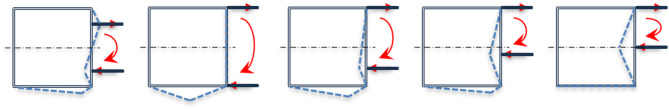
Proposed yield lines considering sidewalls.

In conclusion, the previous findings indicate a significant divergence between the experimentally measured and code-predicted moment capacities for both centric and eccentric DLP connections under out-of-plane moment. The existing codes, which are fundamentally based on yield line theory, assume for the chord face plastification that only the chord face contributes to moment resistance and neglect the potential contribution of the sidewalls—even when the plates are positioned close to them. Based on the experimental tests, the sidewall deformation is minor and not detected for the case of concentric connection with a small β ratio, or eccentric connection with a plate at the mid-depth away from the sidewall. However, the contribution of the sidewall is significant when one plate gets closer or aligned to the bottom sidewall. Accordingly, to treat the convertasim in some design cases, it is proposed that the yield line mechanism shall involve not only the chord face but also the compressed sidewalls. The proposed yield line mechanism is presented in Fig. 25. For the case of eccentric DLP connection with a plate at the face mid-depth, no modification in the yield line is required, and Eqs. 2 & 3 can be applied. For the case of eccentric DLP connection with a plate not at the face mid-depth but going toward the bottom sidewall, Eqs. 7 & 8 can be applied with modification to include yield lines of the compressive sidewall. The inherent stiffness of the SHS corner forces the face and sidewall to deform in coordination to preserve a 90-degree corner angle without distortion. The same shall be applied for the case of centric DLP connection. Equation 1 can be applied with the same modification to include yield lines of the compressive sidewall. For the case of full-height connection, none of the plastification equations is applicable, and other rules for sidewall buckling shall be applied.

## Conclusions and recommendations

A double longitudinal plate connection to transfer out-of-plane bending moment at a T-joint is studied in this paper. The joint is performed from a welded built-up box section for the brace and SHS truss chord. An experimental program is presented to verify relevant design rules and predict capacities from proposed equations, considering three key parameters, namely double-plate spacing, plate width, and connection offset from the chord centerline. The results are summarized as follows:

- The connection behavior is always ductile, and the load keeps increasing, accompanied by large deformation. The connection capacity shall be defined in a way that depends on the deformation, other than by taking the peak load. A face deformation of 3% of the chord depth is used as an indication of the connection capacity before reaching the ultimate load.
- Chord face plastification is the dominant failure mode in most cases. Positioning the plates near the face center (in the middle 75% of the chord depth) makes the connection elastic susceptible to face plastification only with high deformation at small loads.
- Sidewall buckling appears when the compression plate of the double plates gets closer to the chord sidewalls, welded to the chord face at zones outside the middle 75% of the chord depth. At which a large contribution is found from the sidewall, providing greater stiffness and enhancing connection capacity.
- Chord distortion is limited to the full depth connection where both the double plates are welded to the chord sidewalls. Also, little distortion is detected for specimens with 0.75 < β < 1.0, specifically with an upward offset, where one of the double plates is welded to the tension sidewall.
- Increasing the β ratio significantly improves the connection capacity. For β ratios from 0.5 to 0.75 and from 0.75 to 0.875 in the centric connection, the capacity increases by 275% and 170%, respectively. The capacity overall rise reaches 650% from β = 0.5 to 1.00.
- Eccentric connections exhibit lower moment capacities, 50–65% of centric plates around the face center, due to the position of one plate of the DLP near or at face mid-depth.
- The double-plate offset direction upward or downward has the same behavior and connection capacity for β *≤* 0.75. However, for β = 0.875, the downward offset gives 22% higher capacity than the upward offset. It is recommended to avoid welding the compression plate adjacent to the sidewall, but to place it exactly in front of the sidewall.
- The plate width affects the connection capacity positevly, as the capacity increases with the increase of the plate width. Increasing the η ratio from 0.67 to 1.00 results in a moment capacity increase reaching 37%. This magnification decreases to 18% as β increases to 0.875.

### Recommendations

As guidelines for designing DLP connection, a full-depth connection is the first option. If not applicable, center the connection to the chord without offset and maintain a connection depth of at least 75% of the chord depth. If the offset is mandatory, place one of the double plates at the compression sidewall, with the one on the face serving as the tension plate. Use plates with the largest possible width, at least with a width equal to the chord depth. To estimate the DLP connection capacity, two relevant existing cases from the codes can be used. The SHS-to-SHS connection, subject to out-of-plane moment, gives a good prediction, but on the conservative side for the centric DLP connection. These rules shall be modified by considering the yield line of the sidewalls to recover the conservatism. The single longitudinal plate subject to normal force gives a precise prediction for eccentric DLP connection with β = 0.5. For eccentric DLP connection with β other than 0.5, a proposal is suggested in the paper that gives a good prediction, but again, considering the yield line of the sidewall.

It is worth noting that the results in this paper are based on the number of tests performed in the experimental program. Additional tests or numerical studies are required in future works to generalize the results and propose accurate rules for the capacities, also to investigate the effects of extra parameters such as the chord slenderness ratio, plate or weld thickness. Moreover, the research can be expanded to cover the connection behavior under cyclic loadings, as the current results are limited to static load.

## Data Availability

The datasets used and/or analysed during the current study are available from the corresponding author upon reasonable request.

## Abbreviations

ASTM: American Society for Testing and Materials
BUS: Built-up section, performed by welding individual plates
DLP: Double longitudinal plates
HSS: Hollow steel section
LVDT: Linear voltage displacement transducer
SHS: Square hollow section
b_0_: Chord face depth (equal to sidewall width) [mm]
b_1_: Brace depth (presented as the out-to-out distance of the double plates in this paper) [mm]
d: Bolt diameter [mm]
E: Modulus of elasticity of steel [MPa]
E_i_: Internal plastic energy
E: Eccentricity [mm]
F_u_: Ultimate strength of steel [MPa]
F_y_: Yield strength of steel [MPa]
I: Second moment of inertia of a section [mm^4^]
L: Beam span [mm]
l_1_: Brace width (presented as the plate width of the DSLP in this paper) [mm]
M: Out-of-plane bending moment on connection [kN.m]
M_code1_: Moment capacity of SHS-to-SHS connection subject to out-of-plane moment as per design code [kN.m]
M_code2_: Moment capacity of centric single longitudinal plate connection subject to normal force corresponding to P_singlePL_ as per design code [kN.m]
M_proposed_: Moment capacity of eccentric single longitudinal plate connection subject to normal force corresponding to P_singlePLecc_ as proposed in this paper [kN.m]
M_Exp_: Connection moment capacity, the least from M_ult_ and the moment that causes chord face deformation of 0.03b_0_. [kN.m]
M_ult_: Moment corresponding to the maximum load occurs during the test [kN.m]
m_p_: Plastic moment per unit length
n: Number of yield lines
P: Load [kN]
P_singlePL_: Capacity of centric single longitudinal plate connection subject to normal force as per design code [kN]
P_singlePLecc_: Capacity of eccentric single longitudinal plate connection subject to normal force as proposed in this paper [kN]
P_ult_: Maximum load occurs during the test [kN]
t_0_: Chord thickness [mm]
t_1_: Brace thickness (presented as the thickness of DLP in this paper) [mm]
u: Distance between the top sidewall of the chord and the top welded plate [mm]
v: Distance between the chord’s bottom sidewall and bottom welded plate [mm]
β: Brace depth-to-chord face depth ratio (b_1_/b_0_)
Δ_brace_: Deflection of the brace beam at the cantilever tip due to a concentrated load at the beam tip [mm]
Δ_chord_: Deflection of the chord at its midspan due to a concentrated load at the midspan of a fixed–fixed beam [mm]
Δ_D1_: Vertical deflection at the brace tip under the load [mm]
Δ_DLP_: Vertical deflection at the brace tip due to the chord face rotation = sum of the chord face horizontal deformation at tension and compression points [mm]
ε_u_: Ultimate strain [mm/mm]
Φ: Rotation of the yield line
ɳ: Brace width-to-chord face depth ratio (l_1_/b_0_)
${\ell}$: Length of the yield lines
